# Haplotype-resolved Genome of Sika Deer Reveals Allele-specific Gene Expression and Chromosome Evolution

**DOI:** 10.1016/j.gpb.2022.11.001

**Published:** 2022-11-15

**Authors:** Ruobing Han, Lei Han, Xunwu Zhao, Qianghui Wang, Yanling Xia, Heping Li

**Affiliations:** College of Wildlife and Protected Area, Northeast Forestry University, Harbin 150040, China

**Keywords:** Allele-specific expression gene, Sika deer, Chromosome evolution, Structural variation, Rapid antler growth

## Abstract

Despite the scientific and medicinal importance of diploid **sika deer** (*Cervus nippon*), its genome resources are limited and haplotype-resolved chromosome-scale assembly is urgently needed. To explore mechanisms underlying the expression patterns of the allele-specific genes in antlers and the **chromosome evolution** in Cervidae, we report, for the first time, a high-quality haplotype-resolved chromosome-scale genome of sika deer by integrating multiple sequencing strategies, which was anchored to 32 homologous groups with a pair of sex chromosomes (XY). Several expanded genes (*RET*, *PPP2R1A*, *PPP2R1B*, *YWHAB*, *YWHAZ*, and *RPS6*) and positively selected genes (*eIF4E*, *Wnt8A*, *Wnt9B*, *BMP4*, and *TP53*) were identified, which could contribute to **rapid antler growth** without carcinogenesis. A comprehensive and systematic genome-wide analysis of allele expression patterns revealed that most alleles were functionally equivalent in regulating rapid antler growth and inhibiting oncogenesis. Comparative genomic analysis revealed that chromosome fission might occur during the divergence of sika deer and red deer (*Cervus elaphus*), and the olfactory sensation of sika deer might be more powerful than that of red deer. Obvious inversion regions containing olfactory receptor genes were also identified, which arose since the divergence. In conclusion, the high-quality allele-aware reference genome provides valuable resources for further illustration of the unique biological characteristics of antler, chromosome evolution, and multi-omics research of cervid animals.

## Introduction

Cervidae is the second largest family of artiodactyl ruminants (second to Bovidae). As a unique appendage organ of male cervid species (except for the reindeer), the antler grows extremely fast that exceeds even certain cancer tissues [1], [2]. Thus, antler provides an excellent model for studying rapid tissue growth in biological sciences. Sika deer (*Cervus nippon*) is one of the famous cervid animals producing antlers. As of now, there are not many genome resources of sika deer for the study of biology and evolution of Cervidae. Recent studies have reported that expression variation of alleles occurs frequently in mammals [3], [4], [5]. The allelic variants might affect the expression levels of alleles, and the expression variation of alleles is crucial in determining phenotypic diversity [3]. However, as a typical diploid mammal with two homologous chromosome pairs, the allelic variation of cervid species has not been elucidated yet. Therefore, deciphering the genome sequence of each allele chromosome of sika deer is substantial for understanding the expression patterns of allele-specific genes and their phenotypic characteristics (rapid antler growth and inhibition of oncogenesis) [6], [7].

It is generally believed that the variation of the chromosome number and structure is one of the sources of biodiversity. The chromosome number could be increased or decreased by chromosome fission or fusion, respectively [7], [8]. However, the molecular basis and consequence of chromosome variation in mammals remain unsolved [9]. The chromosome number varies dramatically in cervid animals, ranging from 2*n* = 6 (female Indian muntjac) [8] to 2*n* = 70 (such as Siberian roe deer) [10]. Sika deer, as a typical representative animal of the genus *Cervus* in the cervid animals, also has different karyotypes with its closely related species, red deer (*Cervus elaphus*) [11]. However, the concrete mechanism of chromosome evolution between two species still remains to be elucidated. Assembling the phased genome of sika deer contributes to understanding chromosome evolution in cervid animals [12], [13]. It is also crucial to study the biodiversity and social organization of cervid species [14].

In this work, the high-quality Illumina short reads, circular consensus sequencing (CCS) data, and high-throughput chromosome conformation capture (Hi-C) data were generated, which enabled us to assemble a high-quality haplotype-resolved chromosome-level genome of sika deer with the state-of-the-art assembly method. The allele-aware genome of sika deer contributed to exploring the cause of different karyotypes between sika deer and red deer, which would be of help for investigating the expression profiles of alleles in antler and researching the diversity and variation of alleles on homologous chromosomes. Our study also suggested the possible molecular basis for rapid antler growth. Overall, the high-quality genome and annotation information reported here not only investigated differences in expression between alleles on homologous chromosomes, but also provided valuable data and resources for studying structural variations (SVs), the mechanism of chromosome evolution of sika deer, and the molecular basis of rapid antler growth.

## Results

### Haplotype-resolved chromosome-scale genome assembly and annotation of sika deer

**Figure 1:**
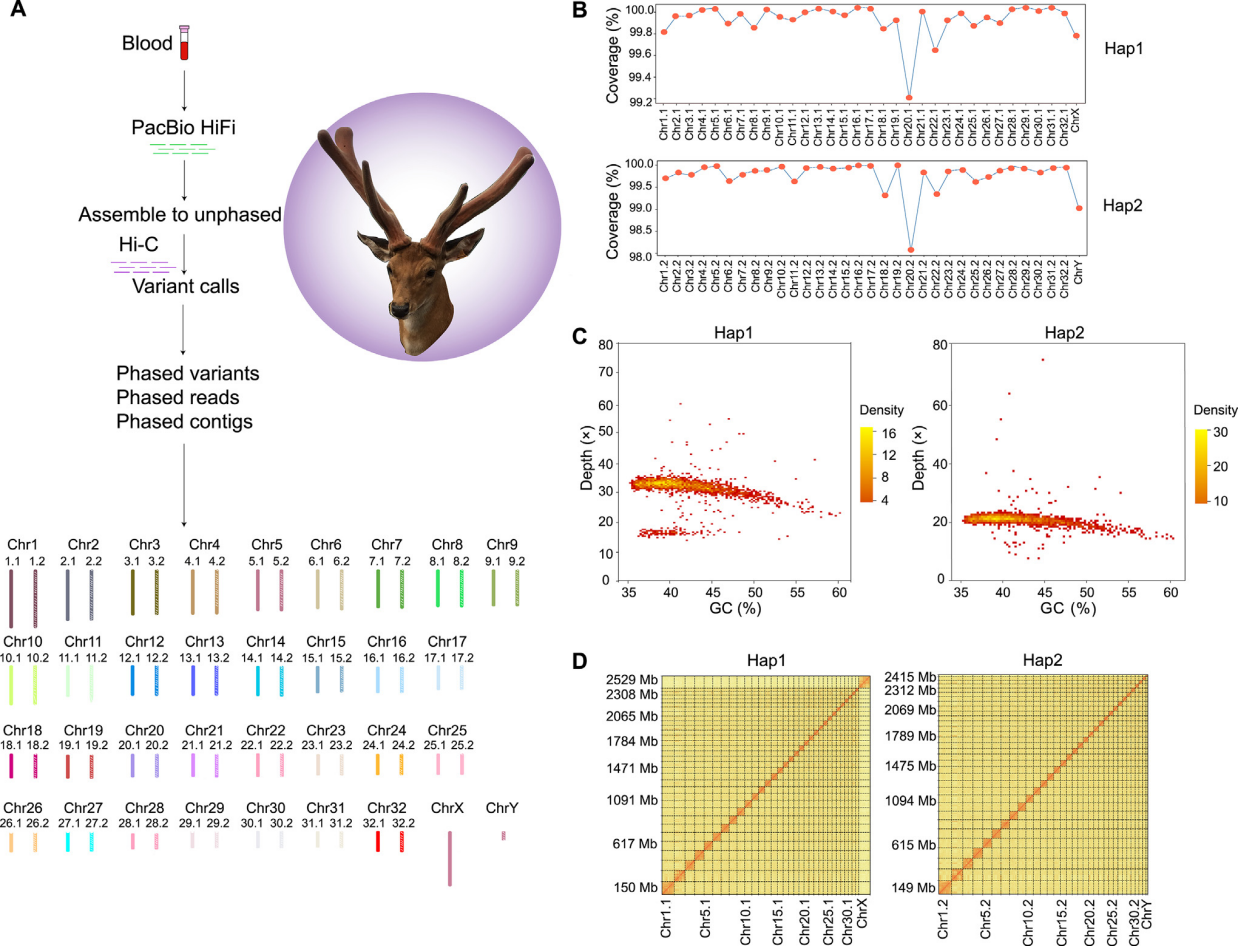
***De novo* assembly and assessment of genome quality** **A.** Overview of the *de novo* assembly of the haplotype-resolved chromosome-level genome of sika deer. **B.** Sequencing coverage of Hap1 and Hap2. **C.** GC depth distributions of Hap1 and Hap2. **D.** Hi-C interaction matrices of Hap1 and Hap2. The diagonal bar represents the frequency of contact between two loci on a chromosome, and the color from light to dark indicates the contact density from low to high. HiFi, high fidelity; Hi-C, high-throughput chromosome conformation capture; Chr, chromosome; Hap1, haplotype 1; Hap2, haplotype 2.

**Figure 2:**
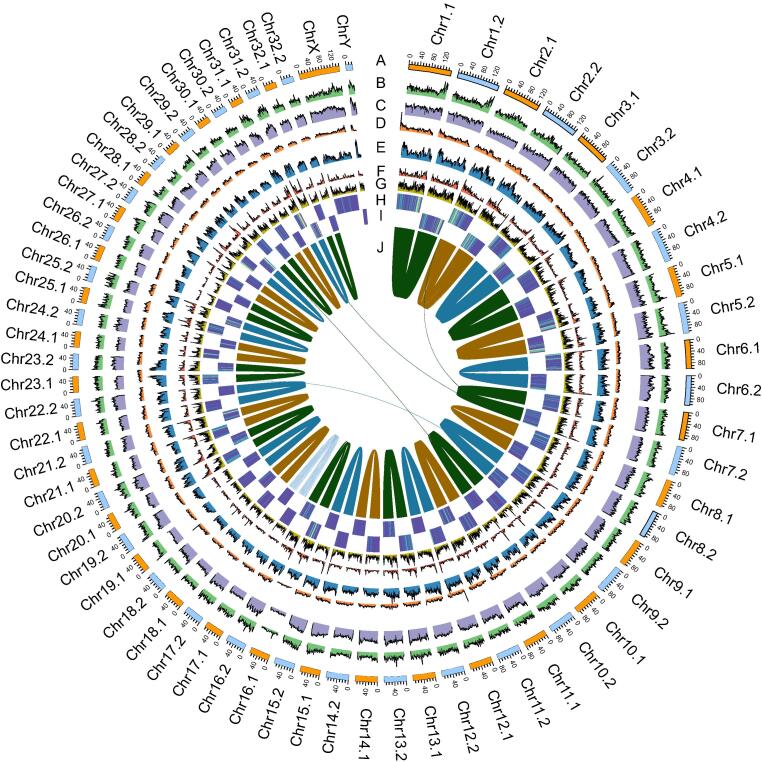
**The overview of haplotype-resolved chromosome-level genome of sika deer** **A.** Length (Mb) of the chromosome. **B.** Density of LTR transposons. **C.** Density of LINE transposons. **D.** Density of DNA transposons. **E.** Density of SINE transposons. **F.** Gene number. **G.** GC content (the windows of 1 Mb). **H.***Ka*/*Ks* of syntenic gene pairs in Hap1. **I.***Ka*/*Ks* of syntenic gene pairs in Hap2. **J.** Links between the core connected alleles. LTR, long terminal repeat; LINE, long interspersed nuclear element; SINE, short interspersed nuclear element; *Ka*, nonsynonymous mutation; *Ks*, synonymous mutation.

A total of 143 Gb (53×–55×) Illumina short reads and 96.4 Gb (36×–37×) PacBio CCS long reads were generated (Table S1). Hifiasm [15], [16] and DipAsm pipeline [7] were used to assemble an accurate allele-aware genome of sika deer (Figure 1A), and the final diploid assembly of sika deer was phased into two haplotypes named “haplotype 1” (Hap1) and “haplotype 2” (Hap2). Hap1 had the size of 2.71 Gb with a contig N50 length of 34.98 Mb, and Hap2 had the size of 2.58 Gb with a contig N50 length of 38.09 Mb (Table S2), indicating the well-resolved haplotype assembly (Figure 1B and C). For improving the haplotype-resolved genome to chromosome scale, approximately 269-Gb (99×–104×) Hi-C paired-end reads were obtained to anchor the contigs into chromosomes. The final assembled monoploid genome of sika deer contained 66 chromosomes, comprising the 32 homologous groups with a pair of sex chromosomes (XY) (Figure 1A and Figure 2). A total of 98.05%–100% of the phased scaffolds were anchored to 66 chromosomes, indicating that most chromosomes were phased correctly (Table S3). The Hi-C interaction matrix of Hap1 and Hap2 also indicated that the chromosome groups were clear cut (Figure 1D). Additionally, Hap1 and Hap2 had 94.5% and 95.0% Benchmarking Universal Single-Copy Orthologs (BUSCO) genes, respectively (Table S4). Compared with the chromosomal-level genome of female sika deer just released recently (Tables S5 and S6) [17], a haplotype-resolved chromosome-level genome of male sika deer assembled in this study had higher contig N50 and higher BUSCO scores, indicating that our assemblies are of high quality.

A total of 22,144 (Hap1) and 18,705 (Hap2) protein-coding genes were predicted in the monoploid genome by a combined strategy of *de novo* gene prediction, homology-based search, and RNA sequencing (RNA-seq). The results of Core Eukaryotic Genes Mapping Approach (CEGMA) showed that 99.57% (Hap1) and 98.28% (Hap2) of genes in the haplotype-resolved genome were predicted, respectively. The mapping ratios of Illumina reads were 99.83% and 98.92% in Hap1 and Hap2, respectively, and the mapping ratios of Expressed Sequence Tag (EST) sequences were both higher than 95% (Table S7). At least 93.80% and 91.60% of protein-coding genes were functionally annotated against databases in Hap1 and Hap2, respectively (Table S8). More than 93.74%–98.37% of the transcripts could be mapped to Hap1, whereas more than 92.22%–97.04% of the transcripts could be mapped to Hap2 (Tables S9 and S10). All the aforementioned results, together with the genome assembly quality standard established by the Vertebrate Genome Project consortium [18], indicate that the allele-aware chromosome-scale genome assembly and annotation of sika deer are of high quality. In addition, non-coding RNAs (ncRNAs) were predicted in the haplotype-resolved chromosome-level genome of sika deer (Table S11).

The allelic chromosome pairs were systematically compared for assessing the differences between the two haplotypes. The results showed that the homologous chromosomes were highly similar with respect to gene number, exon number, intron number, and repeat content (Figure 2; Tables S12 and S13), suggesting that two allelic chromosome pairs of sika deer were functionally equivalent. Nonsynonymous mutation (*Ka*)/synonymous mutation (*Ks*) of syntenic gene pairs also had no considerable difference between the two haplotypes (Figure 2). As the homologous chromosomes of sika deer had a highly similar gene content, Hap1 was used to represent the monoploid sika deer in the following analysis except for a special description. Collectively, the results of multiple approaches revealed the high-quality haplotype-resolved chromosome-scale genome of sika deer.

### Phylogenetic relationship and demographic history of sika deer

**Figure 3:**
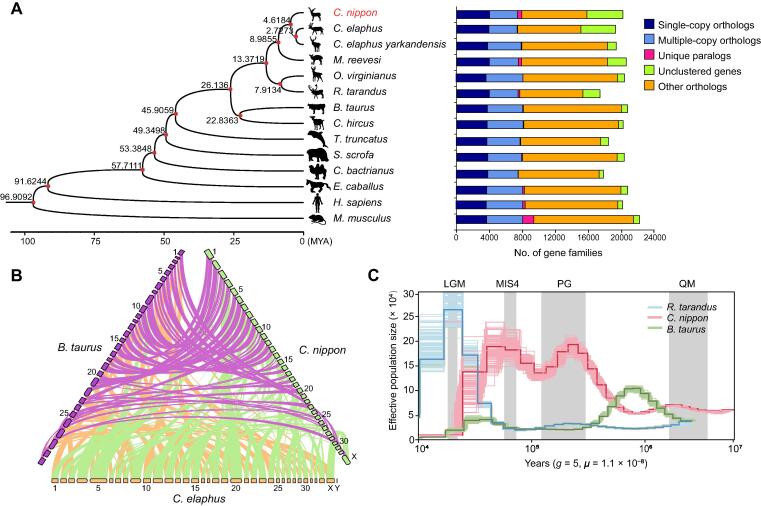
**The phylogenetic relationship, synteny, and population size of sika deer** **A.** The phylogenetic relationship of sika deer and other 13 mammals. The divergence time is shown in the phylogenetic tree. The red nodes indicate that the support values of the branches are 100. The results of gene families are shown in the bar charts on the right side of the phylogenetic tree. **B.** Synteny analysis of sika deer, red deer, and cattle. Purple, green, and orange boxes represent the chromosomes of cattle, sika deer, and red deer, respectively. Different synteny blocks between one species and another are linked by lines of different colors. **C.** The effective population size of sika deer, cattle, and reindeer. “*g*” represents the generation length, and “μ” represents the mutation rate per generation. MYA, million years ago; LGM, Last Glacial Maximum; MIS4, Marine Isotope Stage 4; PG, Penultimate Glaciation; QM, Qingzang Movement.

The sika deer gene model was clustered with the genes from 13 mammals (see Materials and methods). A total of 2665 single-copy homologous genes were identified as shared by 14 mammalian genomes, which were used to construct a phylogenetic tree (Figure 3A). The results showed that the cervid species were closely related to *Bos taurus* and *Capra hircus*, and the common ancestor of cervid species diverged around 26.14 million years ago (MYA). In the cervid species, sika deer was the sister lineage of red deer and Tarim red deer, followed by muntjac, and then white-tailed deer and reindeer. The sika deer also demonstrated overall strong syntenic relationships with red deer and cattle, providing evidence for their phylogenetic relationships (Figure 3B).

To construct and investigate the demographic history of sika deer, the pairwise sequentially Markovian coalescent (PSMC) model was applied to infer the changes in the effective population size (*Ne*) of the ancestral populations of sika deer, reindeer, and cattle. The *Ne* of the ancestral population of sika deer peaked twice at about 0.04 MYA and 0.25 MYA, respectively, whereas it increased sharply in 0.3–0.8 MYA. During the same period, the *Ne* of the ancestral population of cattle gradually decreased. Additionally, the *Ne* of the ancestral population of sika deer dropped two times starting from 0.01–0.04 MYA, and it underwent severe bottlenecks during the Last Glacial Maximum (Figure 3C), providing powerful evidence for the low genetic diversity of the modern population of sika deer [19]. It is probable that the uplift of the Tibetan Plateau led to a sharp decrease in the distribution of sika deer, and the habitat of sika deer has been destroyed to a certain extent by large-scale deforestation caused by humans [20], [21]. Taken together, frequent human activities after the ice age were at least partially responsible for the low genetic diversity of the modern population of sika deer [20], [21].

### Expanded gene families involved in rapid antler growth

To elucidate the biological characteristics and adaptive evolution of sika deer, the gene families between sika deer and aforementioned 13 other mammals were analyzed, revealing that 378 significantly expanded gene families were functionally related to signal transduction (Hippo signaling pathway, PI3K-AKT signaling pathway, and calcium signaling pathway), cell growth and death (cell cycle and apoptosis), and pathways in cancer (Table S14). A total of 78 gene families were identified as significantly contracted, which were significantly enriched in ABC transporters, tight junction, focal adhesion, and ECM-receptor interaction (Table S15). In addition, a total of 42 genes were considered as positively selected genes (PSGs) (Table S16), which were functionally enriched in multiple signal transduction pathways, including MAPK signaling pathway, mTOR signaling pathway, Wnt signaling pathway, and Hippo signaling pathway (Table S17). The previous studies revealed that the PI3K-AKT signaling pathway was critical for the rapid antler growth with inhibition of oncogenesis [22], [23], suggesting that these expanded gene families and PSGs are closely associated with rapid antler growth.

**Figure 4:**
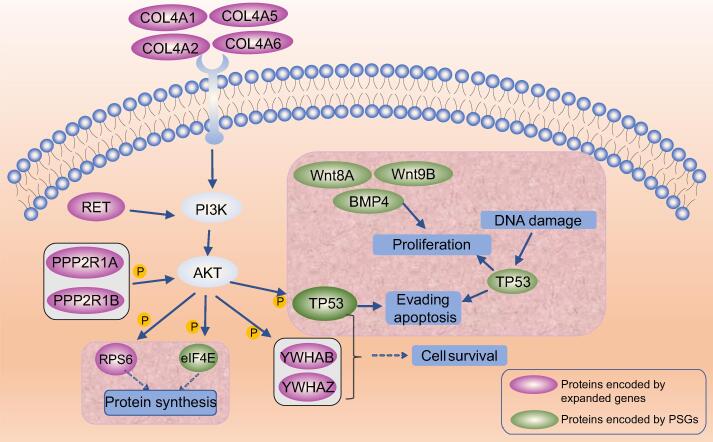
**PI3K-AKT signaling pathway.** Purple indicates protiens encoded by expanded genes in sika deer, and green indicates the proteins encoded by PSGs in sika deer. “P” represents phosphorylation. PSG, positively selected gene.

A total of 10 expanded genes (*COL4A1*, *COL4A2*, *COL4A5*, *COL4A6*, *RET*, *PPP2R1A*, *PPP2R1B*, *YWHAB*, *YWHAZ*, and *RPS6*) and 5 PSGs (*eIF4E*, *Wnt8A*, *Wnt9B*, *BMP4*, and *TP53*) were found to be functionally enriched in PI3K-AKT signaling pathway and related signaling pathway (Figure 4), of which three genes (*YWHAB*, *YWHAZ*, and *RPS6*) responding to DNA damage, cell proliferation, and cell apoptosis were significantly expanded in sika deer [24], [25], [26], [27]. We also observed that six oncogenes were considered as expanded genes (*RET*, *PPP2R1A*, and *PPP2R1B*) or PSGs (*eIF4E*, *BMP4*, and *TP53*). Among them, *PP2A* regulates cell division, cell metabolism, and apoptosis by dephosphorylation of key proteins [28], [29]. It is also important in the regulation of cell growth and proliferation, and the mice with knocked-out PP2Ac α subunit died in the embryonic stage [30], [31]. More importantly, *RET* was significantly expanded in the sika deer compared with the 13 mammals. Previous studies reported that *RET* could regulate cell growth and differentiation by activating several downstream signaling pathways [32], [33], [34]. These results reveal that these genes enriched in the PI3K-AKT signaling pathway and related signaling pathways, together with genes involved in response to DNA damage, cell proliferation, and apoptosis, could coordinately regulate the rapid antler growth and possibly prevent the onset of cancer.

### Genomic SVs and chromosome evolution between sika deer and red deer

**Figure 5:**
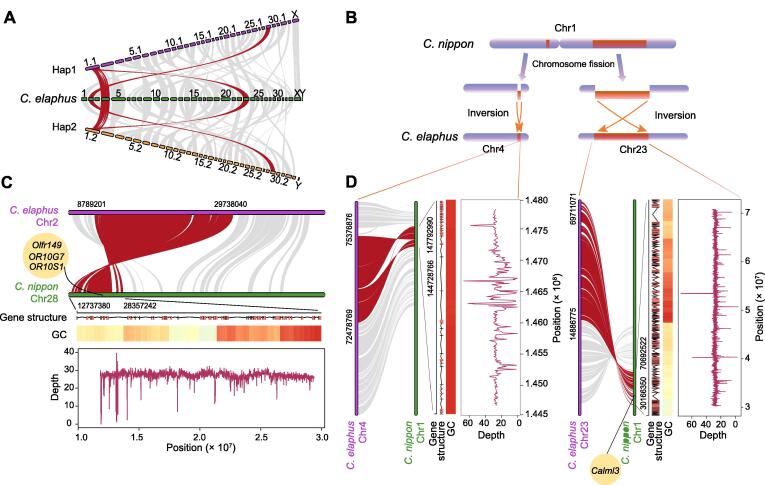
**Chromosome evolution between sika deer and red deer** **A.** Synteny of the haplotype-resolved chromosome-level genome of sika deer and red deer. Synteny blocks between chromosomes of sika deer and red deer are illustrated by red and gray lines, with red lines indicating the inversion regions. **B.** Schematic diagram of divergence between sika deer and red deer. Orange indicates the inversion in the chromosomes. **C.** Synteny of Chr28 in sika deer and Chr2 in red deer. Red indicates the inversion regions. **D.** Synteny of Chr1 in sika deer and Chr4 in red deer, and synteny of Chr1 in sika deer and Chr23 in red deer. Red indicates the inversion regions. The schematic diagram of gene structure shows the distribution of genes in the inversion regions. The heatmap shows the GC content in the inversion regions. The line chart shows the distribution of sequencing depth in the inversion regions.

Overall strong syntenic relationships between diploid sika deer and red deer were observed. It is worth noting that Chr1 of monoploid sika deer had strong syntenic relationships with Chr4 and Chr23 of red deer (Figure 5A), which caused the different karyotypes between sika deer (2*n* = 66) and red deer (2*n* = 68). To further ascertain the mechanism of chromosome evolution between them, the haplotype-resolved genome of sika deer was used for variant calling. Compared with red deer, SVs were found to be distributed on the overwhelming majority of chromosomes in sika deer, among which the inversion was biased toward Chr1 and Chr28 of sika deer, accounting for 29% and 32% of the total length of Chr1 and Chr28, respectively. All these results indicated that there existed inversions during the divergence between Chr1 of sika deer and Chr4 and Chr23 of red deer. Additionally, inversions were also observed in Chr28 of sika deer and Chr2 of red deer (Figure 5B–D).

To test whether these inversion regions were associated with specific functions, the genes distributed in Chr1 inversion regions of sika deer and corresponding regions of red deer were identified, respectively. Kyoto Encyclopedia of Genes and Genomes (KEGG) enrichment analysis was performed on the genes that were located in the Chr1 inversion region of sika deer, which were mainly enriched in some signaling pathways related to biosynthesis (folate biosynthesis, ovarian steroidogenesis, and steroid hormone biosynthesis) and metabolism (metabolic pathways and arachidonic acid metabolism) (Table S18). Compared with the gene sets that were distributed in the Chr1 inversion region of sika deer, a total of 78 genes were lost in Chr4 and Chr23 of red deer. These 78 genes were also searched on National Center for Biotechnology Information (NCBI; https://www.ncbi.nlm.nih.gov/) to reduce the false-positive results. It turned out that the Chr4 and Chr23 of red deer lost 33 genes compared with Chr1 of sika deer, inferring that the genome quality of red deer was not sufficient for investigating all genes. We further observed that 16 genes on Chr1 of sika deer distributed on multiple chromosomes of red deer, such as Chr10, Chr11, and Chr29, revealing that these 16 genes might be translocated during the divergence between sika deer and red deer. Finally, Chr4 and Chr23 in red deer uniquely lost 17 genes, one of which was enriched in olfactory transduction (*Calml3*) (Figure 5D).

The Chr28 in two haplotypes of sika deer showed high homology with Chr2 of red deer (Figure 5C). The genes distributed in the inversion regions of Chr28 in sika deer and corresponding regions of Chr2 in red deer were identified, respectively. KEGG enrichment analysis was performed on the gene sets that were distributed in the Chr28 inversion regions of sika deer, which were aligned in the p53 signaling pathway, HIF-1 signaling pathway, glycolysis/gluconeogenesis, propanoate metabolism, and pyruvate metabolism (Table S19). Compared with the gene sets that were distributed in the Chr28 inversion regions of sika deer by the aforementioned methods, seven genes were translocated, and six genes were lost in Chr2 of red deer. Among them, three genes were enriched in olfactory transduction (*Olfr149*, *OR10G7*, and *OR10S1*) (Figure 5C), suggesting that the olfactory sensation of sika deer might be more powerful than that of red deer. Overall, our results cast a novel light on the mechanism of chromosome evolution between sika deer and red deer.

**Supplementary Figure S1:**
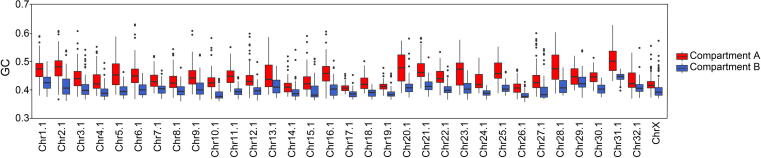
**Boxplot of GC content in genomic compartment A/B** The horizontal line inside the box is the median. The black dot indicates the outlier.

**Supplementary Figure S2:**
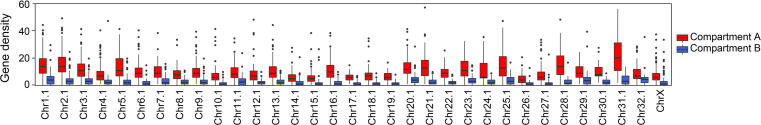
**Boxplot of gene density in genomic compartment A/B** The horizontal line inside the box is the median, and the black dot shows the outlier.

**Supplementary Figure S3:**
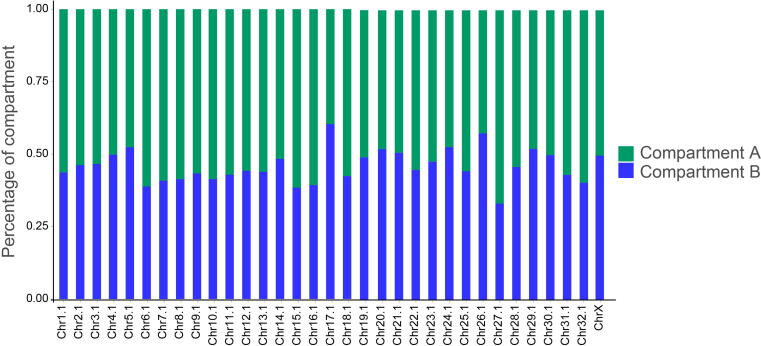
**The distribution of compartment A/B on the chromosome of the sika deer genome**

To further explore the impact of chromosome evolution on three-dimensional (3D) chromatin architectures in sika deer, we performed the 3D chromatin architecture analysis on the sika deer genome, including compartment A/B and topologically associated domains (TADs). The compartment A/B was identified at 500-kb resolution. As in previous studies [9], the results also showed that compartment A had higher GC content and gene density than compartment B (Figures S1 and S2). In addition, compartment A and compartment B were randomly distributed on all chromosomes without bias (Figure S3), speculating that compartment A/B may have little effect on chromosome evolution during the divergence between sika deer and red deer.

**Supplementary Figure S4:**
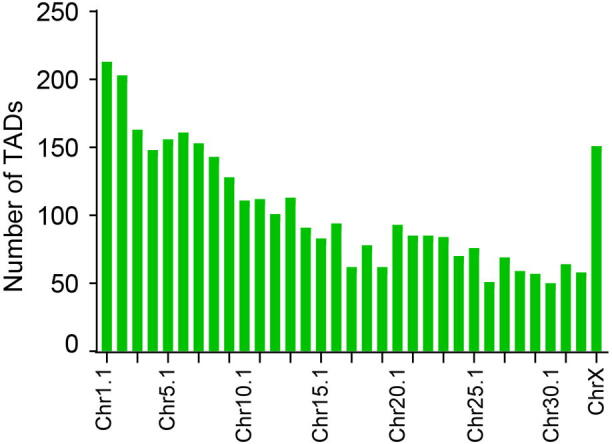
**Distribution of TADs on chromosomes in the sika deer genome at 40 kb resolution** TADs, topologically associated domains.

**Supplementary Figure S5:**
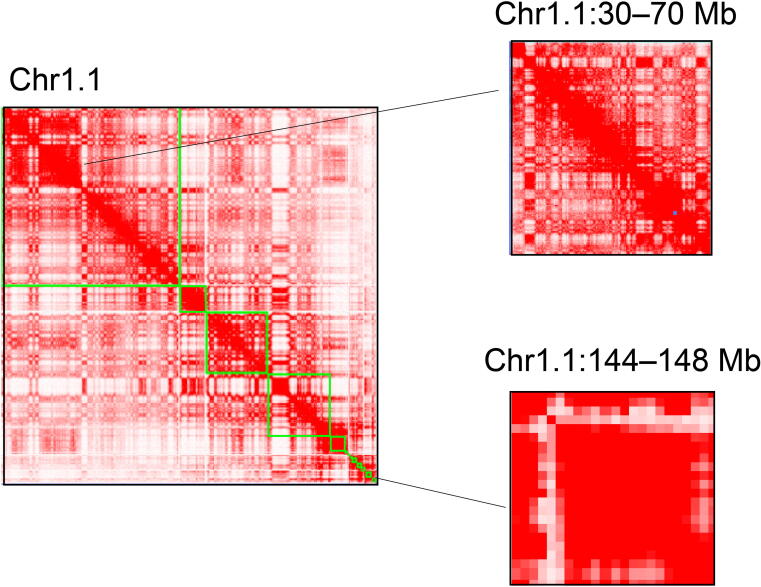
**Hi-C contact heatmaps of Chr1.1 in the haplotype-resolved genome of sika deer** The left figure shows the Hi-C contact heatmaps of Chr1.1, and the right figure shows the Hi-C contact heatmaps of the inversion regions on Chr1.1.

**Supplementary Figure S6:**
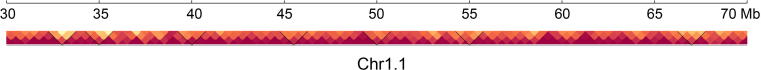
**Identification of TADs in 30–70 Mb on Chr1 in sika deer genome at 500 kb resolution** The threshold used for the identification of TAD is 0.001. The color from light to dark indicates the interaction strength from low to high.

**Supplementary Figure S7:**
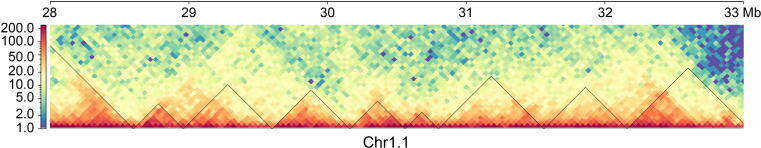
**Identification of TADs in 28–33 Mb on Chr1 of sika deer genome at 40 kb resolution** A threshold of 0.005 was used for the identification of TADs, and the color from blue to red indicates the interaction strength from low the high.

**Supplementary Figure S8:**
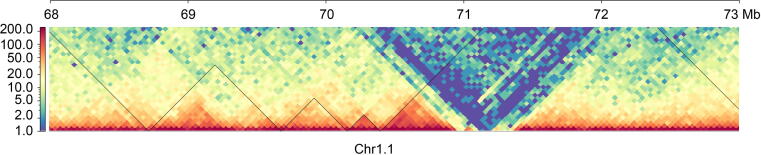
**Identification of TADs in 68–73 Mb on Chr1 of sika deer genome at 40 kb resolution** A threshold of 0.005 was used for the identification of TADs, and the color from blue to red indicates the interaction strength from low the high.

**Supplementary Figure S9:**
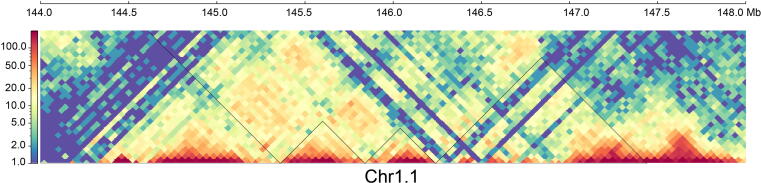
**Identification of TADs in 144–148 Mb on Chr1 of sika deer genome at 40 kb resolution** A threshold of 0.005 was used for the identification of TADs, and the color from blue to red indicates the interaction strength from low the high.

TADs were considered as fundamental units of 3D eukaryotic genome organization. In the present study, a total of 3427 self-interacting regions were identified at 40-kb resolution, with an average length of 713 kb. There is no correlation between the percentage of TADs on the chromosome and the length of the chromosome. However, the large chromosomes generally had more TADs than the short ones (Figure S4). In addition, the Hi-C contact heatmap of Chr1 was identified, including Hi-C contact heatmaps of two inversion sites (Figure S5). TADs were also identified at the sites of inversion regions in Chr1. The results showed that the TADs were scattered in the inversion regions of 30–70 Mb at 500-kb resolution (Figure S6). TADs were identified at both ends of the inversion regions to further understand the chromosome evolution in sika deer at 40-kb resolution. Multiple consecutive TADs were found in 28–33 Mb and 68–73 Mb (Figures S7 and S8). TADs were also identified in another inversion regions (144–148 Mb), and several consecutive TADs were detected (Figure S9). However, this does not mean that the chromosome evolution between sika deer and red deer was greatly affected by TADs.

### Expression pattern of alleles during the sika deer antler growth

**Figure 6:**
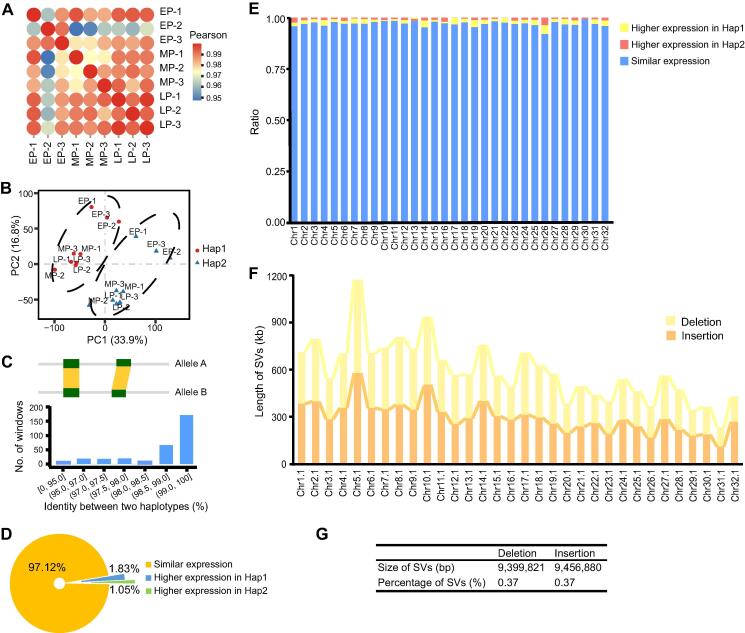
**Haplotype comparison of diploid sika deer** **A.** Correlation heatmap of transcriptome data in Hap1. EP indicates the stage of antler growing to a saddle-like appearance; MP indicats the stage of antler growing with two branches; LP indicates the stage of antler growing with three branches. **B.** PCA of allele expression profiles of sika deer antler at three developmental periods. **C.** Comparison of two haplotypes using 10-Mb nonoverlapping windows. **D.** Statistics of alleles. **E.** Distribution histogram of alleles on the chromosomes. **F.** Distribution histogram of SVs between haplotypes on chromosomes. **G.** Statistics of SVs between two haplotypes. PCA, principal component analysis; PC, principal component; SV, structural variation.

**Supplementary Figure S10:**
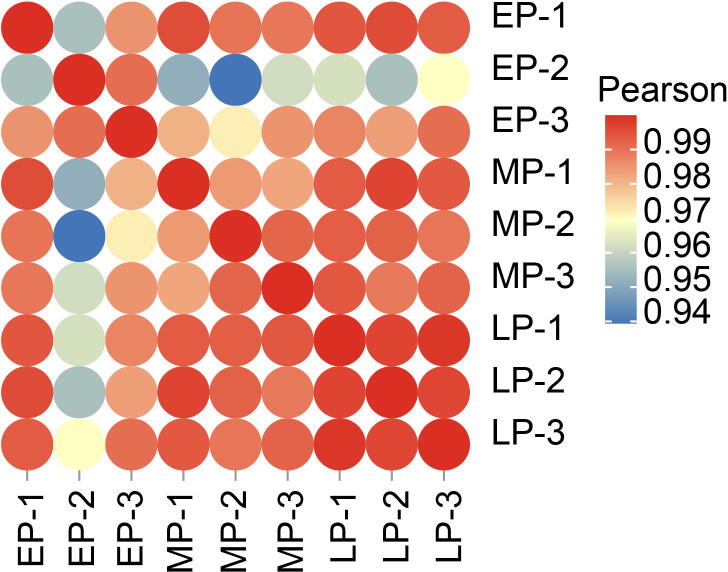
**Correlation heatmap of transcriptome data in Hap2** The color of the circles from blue to red indicates the correlation from low to high between different samples.

Analysis of transcriptome data using one haploid genome as a reference genome could miss allele-specific expression (ASE) or novel expression patterns [35]. Therefore, the gene expression profiles of antlers were reanalyzed using our previous transcriptome data generated from three different periods that represented the whole antler development (BioProject: PRJNA552158) [36] (Figure 6A, Figure S10). The results of principal component analysis (PCA) of the monoploid genome of sika deer indicated that the antler transcriptomes were mainly shaped by genome-wide ASE, followed by the expression of genes at different developmental periods (Figure 6B). Additionally, the haploid genome sequences were aligned stringently for understanding the sequence divergence of two haplotypes, showing the 99.6% sequence identity (Figure 6C).

A total of 12,534 reliable homologous genes were identified in allelic chromosome pairs by combining the synteny and coordinate strategies (Table S20). The vast majority of alleles (97.12%) were found to be coordinately expressed in the two haplotypes during the rapid antler growth (Figure 6D), suggesting that the expression is generally not biased between the two haplotypes and most alleles have similar functions in regulating the rapid antler growth. The alleles in antler were also discovered to distribute on 32 homologous chromosomes without any bias (Figure 6E), revealing that the ASE genes were distributed randomly throughout the sika deer genome. Additionally, the phased diploid genome facilitated the detection of SVs between two haplotypes, including deletion and insertion. The SVs were distributed on 32 autosomes of sika deer (Figure 6F; Table S21) and spanned 18.9 Mb, representing almost 0.74% of the haploid genome (Figure 6G).

**Supplementary Figure S11:**
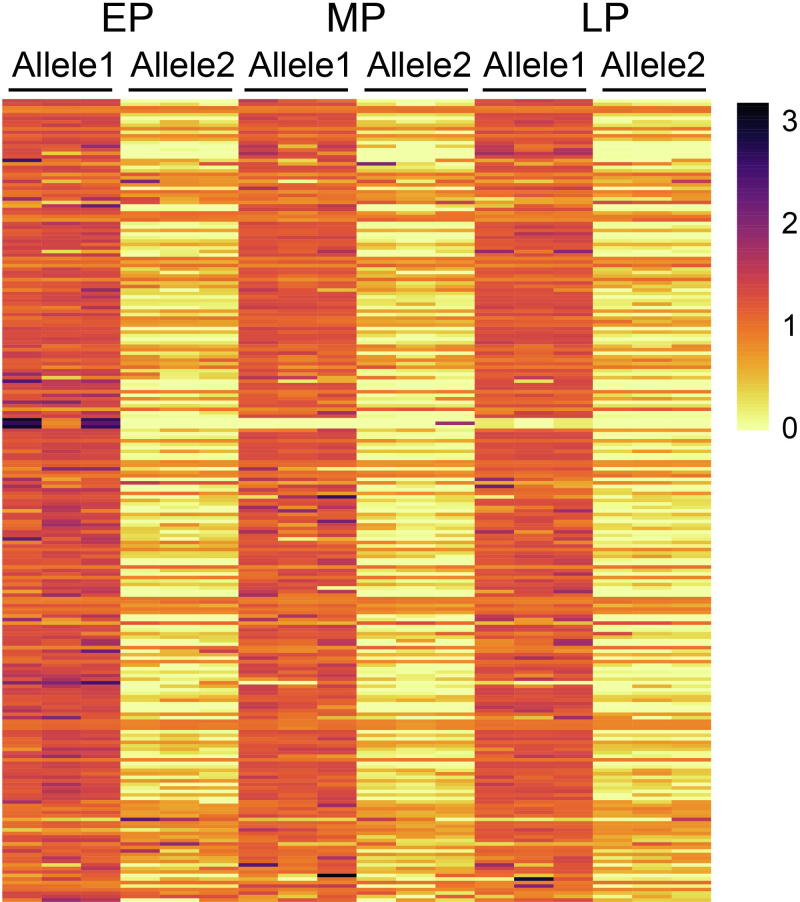
**Expression heatmap of allele-specific expression genes biased to Hap1** The color from yellow to red indicates the expression level from low to high.

**Supplementary Figure S12:**
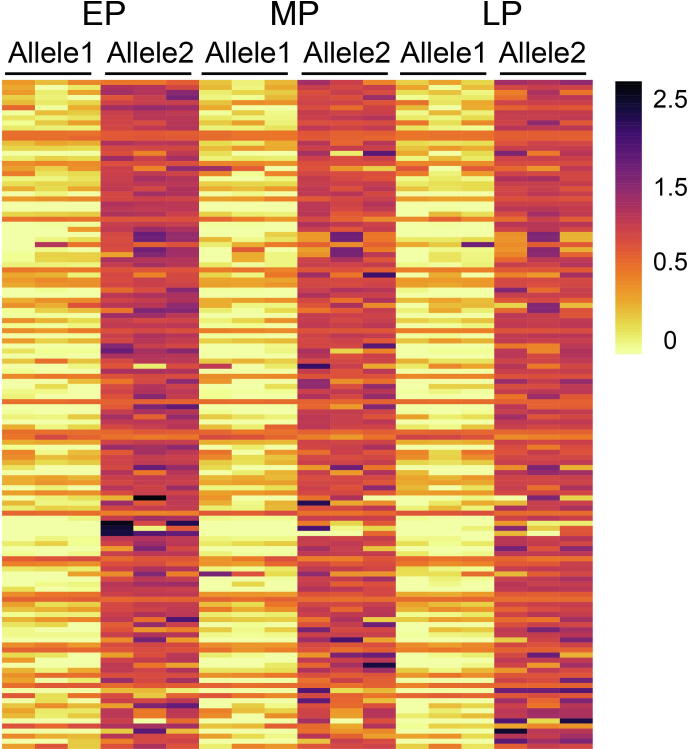
**Expression heatmap of allele-specific expression genes biased to Hap2** The color from yellow to red indicates the expression level from low to high.

**Supplementary Figure S13:**
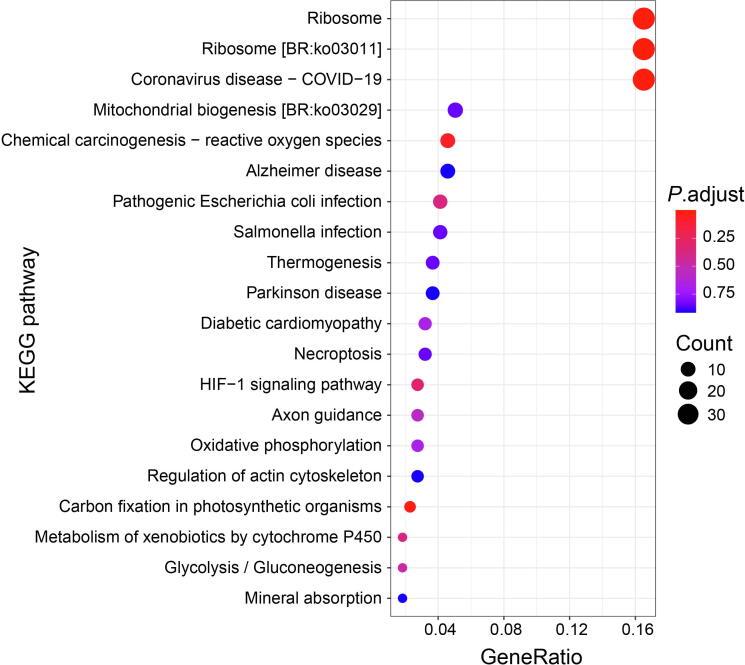
**KEGG enrichment analysis of the allele-specific expression genes**

**Supplementary Figure S14:**
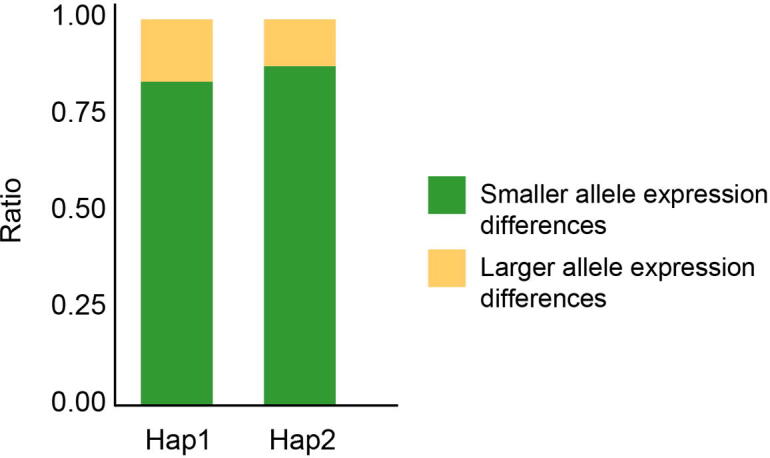
**Percentage of each category of allele expression bias in the two haplotypes**

The ASE genes were investigated in the haplotype-resolved chromosome-level genome of sika deer without the parental information, which were in allele imbalance between two haplotypes. Only 2.9% (361) of homologous genes were regarded as ASE genes with the expression biased toward a single haplotype, of which the expression of 229 ASE genes was biased toward the Hap1 (Figure S11) and the expression of 132 ASE genes was biased toward the Hap2 (Figure S12). These ASE genes showed functional enrichment in multiple biological processes, including ribosome, HIF-1 signaling pathway, axon guidance, mitochondrial biogenesis, metabolism of xenobiotics by cytochrome P450, and chemical carcinogenesis-reactive oxygen species (Figure S13). There were some oncogenes obviously biased toward Hap1 or Hap2, such as *RPLP1*, *RPL3*, *RPS10*, *RPL10*, *RPL23a*, *SLC7A3*, *COL2A1*, and *PEBP1*, indicating that the alleles might interplay to regulate rapid antler growth. In addition, based on the differential expression patterns observed in two haplotypes, we defined the smaller allele expression differences with 2 < |log_2_ fold change (FC)| < 8 (*P* < 0.05) and larger allele expression differences with |log_2_ FC| > 8 (*P* < 0.05). Results showed that the allele expression differences of diverse categories were relatively stable in two haplotypes (Figure S14).

## Discussion

The genetic research of sika deer and related development efforts have not been commensurate with its importance due to the scarcity of genome resources. Currently, we decode the first high-quality haplotype-resolved chromosome-scale genome of diploid sika deer by combining the new sequencing technology and Hi-C scaffolding, which is critical for studying the role of variation in genome function and phenotype [37]. We constructed the expression profile of alleles in the sika deer antler, and proposed the possible molecular basis for rapid antler growth. Our study also contributes to research on the chromosome evolution of cervid animals, especially the mechanism of chromosome evolution between sika deer and red deer. In summary, this high-quality monoploid genome is of sufficient quality for exploring the biological characteristics of cervid species and future multi-omics research of sika deer.

In the present study, the identification of the Y chromosome of sika deer undoubtedly fills the gap in reference genome resources of male sika deer. The genome of male sika deer is beneficial to genomic selection breeding, which is also helpful to retain the germplasm resources with higher antler yields. More importantly, two haplotype genomes of sika deer could more accurately and completely reflect its genetic information, which provides a more complete reference genome for the study of sika deer. The benefit of the haplotype-resolved chromosome-level genome of sika deer also includes that it can be used to explore the expression of alleles and the differences in some phenotypic characteristics of sika deer [38]. This has been confirmed by many haplotype-resolved genomes published recently, such as the haplotype-resolved genomes of plants have filled an important gap in exploring their unique traits using ASE analysis [15], [39]. Additionally, as a secondary sex characteristic of male cervid animals, male cervid species annually grow deciduous antlers. Thus, comparing the diversity of alleles between the two haplotypes of sika deer contributes to understanding the potential molecular basis of male antler growth, and it is important to study the molecular mechanism of the unique biological characteristics of antlers.

In the genus *Cervus* of cervid animals, the variation of chromosome number is dominated by the variation of autosome number, of which chromosome fission is considered to be the main molecular mechanism [11]. In our study, the results revealed that Chr1 of monoploid sika deer showed strong syntenic relationships with Chr4 and Chr23 of red deer, inferring that the sika deer (2*n* = 66) may diverge into red deer (2*n* = 68) through chromosome fission. The results of SVs between sika deer and red deer revealed that inversion played a dominant role in this process, providing a novel perspective to understand the mechanism of chromosome evolution in cervid animals. However, we cannot further determine the chromosome evolution between sika deer and red deer by comparing the 3D chromatin architectures due to the unavailability of Hi-C data of red deer. Additionally, red deer was found to uniquely lose three genes associated with olfactory by comparing the gene sets in the inversion regions of sika deer and red deer. A previous study also analyzed the adaptive evolution of olfactory-related genes in cervid animals using comparative genomics [40]. Our study provides a further solid foundation and detailed reference for the studies of the adaptive evolution of olfactory-related genes in cervid species.

The role of alleles on homologous chromosome pairs in cervid animals has long been overlooked. The phased haplotype-resolved genome of sika deer facilitated the interpretation of the expression patterns and functions of alleles. In the present study, the alleles generally had no notable differential expression between two haplotypes, whereas differential gene expression was still observed in a few ASE genes, which was potentially associated with multiple biological processes. We found that some genes showing ASE were involved in oncogenesis. However, whether this means that one of the haplotypes plays a more important role during the rapid antler growth without carcinogenesis requires further experiments.

A recent study reported that the expression of proto-oncogenes was vital for antler fast growth [23], and the expression of several tumor suppressor genes was necessary for antler growth and inhibition of oncogenesis [23], [41]. We found that several proto-oncogenes and tumor suppressor genes were identified as the expanded genes or PSGs in the sika deer lineage, which were assigned to the PI3K-AKT signaling pathway and related pathways. It has been postulated that the PI3K-AKT signaling pathway was involved in regulating cell proliferation, differentiation, migration, and cell-cycle progression [42], and it was one of the crucial pathways regulating the initiation, development, and regeneration of antler [42], [43], [44], [45]. Thus, we inferred that the multiple copies of *RET* in sika deer might enhance the rapid antler growth through interaction with the PI3K-AKT signaling pathway. *TP53* has one copy and it was identified as a PSG in the sika deer lineage, which strengthens the insight that cervid species may have evolved an enhanced p53 signaling pathway for an efficient cancer-defense mechanism [46]. The rapid antler growth is a complex process regulated by multiple factors, and these expanded genes and PSGs (*RET*, *PPP2R1A*, *PPP2R1B*, *eIF4E*, *BMP4*, and *TP53*) might play a pivotal role in this process. These cancer-related genes might coordinately regulate the rapid antler growth, and form the unique cancer defense mechanism of cervid species. To thoroughly elucidate the molecular mechanism of antler rapid growth and defend against cancer in cervid species, other haplotype-resolved chromosome-scale genomes of cervid species and further functional experiments are required in the future. In summary, our haplotype-resolved chromosome-scale genome of sika deer offers a holistic view of its expression profiles, functions of alleles, and chromosome evolution of cervid species. It will also be helpful for studying the therapy of cancer in humans.

## Conclusion

In the present study, we provided a haplotype-resolved chromosome-scale genome, which is, to our knowledge, the first high-quality available diploid genome of sika deer. This allowed us to explore the ASE patterns between the two homologous chromosomes. Most alleles were found to be co-expressed in the rapidly growing antlers, while at the same time preventing the onset of cancer. Several expanded genes or PSGs (*RET*, *PPP2R1A*, *PPP2R1B*, *YWHAB*, *YWHAZ*, and *RPS6*) were also considered to play a key role in this process. In addition, our results revealed that chromosome fission might occur during the divergence of sika deer and red deer, which resulted in an increase in the chromosome numbers of red deer. Overall, our study will promote the research of the unique characteristics of antler (rapid growth and low cancer rate) and the chromosome evolution in cervid species. It also provides valuable resources and references for multi-omics studies of sika deer.

## Materials and methods

### Sample collection

A fresh blood sample was collected from a 7-year-old healthy male sika deer in Jindi Deer Industry Co. Ltd. (Ear tags: 1220; Harbin, Heilongjiang Province, China), whose parents were both purebred sika deer. DNA was extracted from the fresh blood sample for constructing the libraries.

### Library construction and sequencing

An Illumina paired-end library was constructed using the TruSeq Nano DNA HT Sample Preparation Kit (Catalog No. TG-202-1003, Illumina, San Diego, CA) according to the manufacturer’s instructions, and sequenced by Illumina NovaSeq 6000 (Illumina, San Diego, CA). Three paired-end CCS libraries with the small insert size of 15 kb were constructed, and sequenced using the PacBio Sequel II platform (PacBio, San Francisco, CA). High-fidelity (HiFi) reads were generated by CCS software (https://github.com/PacificBiosciences/ccs). To improve the allele-aware genome to chromosome scale, two Hi-C libraries were constructed and sequenced by Illumina NovaSeq 6000 using DNA extracted from the same individual.

### Genome assembly

Haplotype-resolved genome of sika deer was assembled using Hifiasm [15], [16] with the default parameters. DipAsm pipeline [7] was applied to combine HiFi reads and Hi-C raw data. The haplotype-resolved chromosome-level genome was assembled as follows: (1) Hifiasm was first used to obtain an unphased assembly. Juicer (v1.5) [47] and a 3D *de novo* assembly (3D-DNA; v180922) [48] were used to scaffold the contigs. (2) DeepVariant (v0.8.0) [49] was used to perform small variants, and Hi-C reads were mapped to the scaffolds. (3) HapCUT2 (v1.1) [50] was used to generate sparse phasing at the chromosome scale. (4) WhatsHap (v0.18) [51] was used to generate fine-scale chromosome-long phasing by combining the haplotypes with PacBio HiFi data. (5) To reference-assisted scaffolding, minimap2 (v2.17) [52] was used to align contigs. (6) Purge_dups pipeline [53] was used to remove haplotig sequences from the initial assembly genome. (7) HiC-Pro was used to align Hi-C reads to contigs, and 3D-DNA was applied for correcting misassembles, anchor, order, and orient fragments of DNA. Juicebox Assembly Tools (v1.9.9) was used to manual correction connections. (8) Juicebox [47] and plotHicGenome (v0.1.0) were used to analyze and visualize Hi-C-assembled scaffolds. (9) Y chromosome of the sika deer was detected using the Diamond (v0.9.10) based on the previous study of the red deer genome.

### Genome annotation

#### Gene prediction

The completeness and accuracy of the genome assembly of sika deer were evaluated based on BUSCO, and the transcriptome was mapped to the genome to assist in verifying the genome quality. Illumina short reads were mapped into the haplotype-resolved genome of sika deer by Burrows–Wheeler Aligner (BWA) for estimating the accuracy of genome assembly, and CEGMA software was also used to estimate the quality of the genome assembly. In addition, 2743 EST sequences of sika deer were downloaded from NCBI and aligned against the two haplotype-resolved genomes to further verify the quality of the genome assembly.

Protein-coding genes were annotated by integrated evidence from the homology-based search, *de novo* prediction, and transcriptome data. Briefly, the homologous gene sets of cattle, red deer, sheep, mouse, and reindeer were downloaded from NCBI for homology-based prediction. *De novo* prediction was performed by AUGUSTUS (v3.0.3) [54] and SNAP [55]. We also downloaded the transcriptome data of multiple organs of sika deer from NCBI, including heart (SRA: SRS3900676), liver (SRA: SRS3900672), spleen (SRA: SRS3900692), lung (SRA: SRS3900690), and kidney (SRA: SRS3900689).

Gene function annotation was then performed by aligning against multiple public databases, including Non-Redundant Protein Sequence Database (NR), Gene Ontology (GO), InterProScan, Swiss-Prot, Translation of EMBL (TrEMBL), KEGG, and Clusters of orthologous groups for eukaryotic complete genomes (KOG). In addition, the haplotype-resolved genome was aligned against the Rfam database and vertebrate ribosomal RNA (rRNA) database to predict ncRNAs, including small nuclear RNA (snRNA), microRNA (miRNA), and rRNA.

#### Repeat annotation

The homology-based search and *ab initio* method were both used to annotate repetitive sequences of the sika deer genome. To identify the types of repeat elements, a transposable element library was constructed by Tandem Repeat Finder (TRF) [56], LTR_FINDER [57], and RepeatModeler [58]. The known repeat element was searched against the Repbase database using RepeatMasker [57] and RepeatProteinMask [59].

### Construction of phylogenetic tree and estimation of divergence time

TreeFam (v4.0) [60] was used to construct gene families in the six cervid animals (sika deer, red deer, white-tailed deer, reindeer, Tarim red deer, and muntjak) and eight other mammals (cattle, sheep, human, horse, mouse, pig, camel, and dolphin). Based on the aforementioned results, CAFÉ (v4.2) [61] was used to determine the gene family expansion and contraction. KEGG enrichment analysis was implemented for expanded and contracted gene families in sika deer. To construct the phylogenetic tree, the single-copy genes shared within 14 mammalian genomes were identified.

The divergence time among different species was calculated based on fossil evidence. MCMCtree from the PAML (v4.8) [62] package was applied to estimate the divergence time with default parameters. The PSMC [63] model was used to infer the demographic history of sika deer with the parameter: psmc -N25 -t15 -r5 -p “4+25*2+4+6”.

### Identification of PSGs

To identify the PSGs of sika deer, the high-confidence single-copy orthologous genes were identified based on the 14 mammals mentioned above. MUSCLE (v3.8.31) was used to conduct the multiple sequence alignment. The branch-site model was first selected to determine the PSGs, and the likelihood ratio test (LRT) in the CodeML of PAML was then used to detect the PSGs with the sika deer lineage as the foreground branch. *P* value was calculated by chi-square statistic and corrected by the Benjamini–Hochberg method. The genes with *P* < 0.05 were considered as PSGs. The PSGs obtained were subjected to KEGG enrichment analysis using the clusterProfiler package [64].

### SVs between sika deer and red deer

Genomic SVs, detected by PacBio CCS reads, provided more convenient conditions for studying polymorphic variations, chromosome evolution [65], cancer research [66], and phenotypes in organisms [67], [68], [69], [70]. The haplotype-resolved genome was beneficial for better interpreting the SVs. To understand the SVs between the sika deer and its sister lineage (red deer), MCScanX [71] was used to perform synteny analysis. Sniffles [72] was then used to identify SVs between sika deer and red deer, including inversion, deletion, duplication, and insertion.

3D chromatin architecture was performed on sika deer at two different hierarchical levels using HiCExplorer (https://training.galaxyproject.org/training-material/topics/epigenetics/tutorials/hicexplorer/tutorial.html), including compartment A/B and TADs. HicPCA was used to calculate compartment A/B, and hicFindTADs program was used to call TADs at 40-kb resolution in the sika deer genome.

### Allele identification and haplotype comparison

The alleles were identified by combining synteny, coordinate, and the mapping ratio. Blastn was used to perform the multiple sequence alignment between the two haplotype genomes. MCScanX and MUMmer were used to define the synteny blocks between Hap1 and Hap2. Accordingly, the paired genes (identity > 97%) with high similarity in each synteny block were considered as reliable alleles A and B.

The phased monoploid genome was helpful for understanding the expression profile of alleles in the antlers of sika deer. In the present study, HISAT2 was used to map the RNA-seq reads that were sequenced from the mesenchymal tissue of the sika deer antler [36] to Hap1 and Hap2, respectively. The expression level of each transcript was estimated with HTSeq. The differences in expression between alleles were estimated by DESeq2 [73]. To ensure the accuracy and reliability of the results of ASE genes, the genes with the count of 0 in all samples from two haplotypes were filtered out. The homologous genes that meet |log_2_ FC| > 2 and *P* < 0.05 were defined as ASE genes. KEGG enrichment analyses of ASE genes were implemented by the clusterProfiler package in R. Additionally, Assemblytics [74] was used to identify SVs between haplomes.

## Ethical statement

All experimental designs and animal handling were approved by the Institutional Animal Care and Use Committee of Northeast Forestry University, China (Approval No. 2022049).

## Data availability

The raw sequencing data generated in this study have been deposited in the Genome Sequence Archive [75] at the National Genomics Data Center (NGDC), Beijing Institute of Genomics (BIG), Chinese Academy of Sciences (CAS) / China National Center for Bioinformation (CNCB) (GSA: CRA007487), and are publicly accessible at https://ngdc.cncb.ac.cn/gsa. The whole-genome sequence data reported in this study have been deposited in the Genome Warehouse [76] at the NGDC, BIG, CAS / CNCB (GWH: GWHBJVV00000000 and GWHBJVU00000000), and are publicly accessible at https://ngdc.cncb.ac.cn/gwh.

## Competing interests

The authors have declared no competing interests.

## CRediT authorship contribution statement

**Ruobing Han:** Resources, Writing – original draft, Data curation, Investigation, Writing – review & editing, Visualization, Formal analysis. **Lei Han:** Data curation, Visualization, Investigation, Formal analysis, Writing – review & editing. **Xunwu Zhao:** Writing – review & editing. **Qianghui Wang:** Writing – review & editing, Formal analysis. **Yanling Xia:** Resources. **Heping Li:** Funding acquisition, Conceptualization, Supervision, Project administration, Resources. All authors have read and approved the final manuscript.

## Acknowledgments

We would like to thank the National Key R&D Program of China (Grant No. 2018YFC1706601) and the Natural Science Foundation of Heilongjiang Province of China (Grant No. C2017012). We thank Xianlan Cui for the language editing of this manuscript.

## Supplementary material

Supplementary Table S1 **The statistics of PacBio sequencing data**

Supplementary Table S2 **Summary of the haplotype-resolved genome of sika deer**

Supplementary Table S3 **Statistics information of chromosome-level of the haplotype-resolved genome**

Supplementary Table S4 **Summary of BUSCOs recovered in the haplotype-resolved genome of sika deer**

Supplementary Table S5 **Summary of comparison with the recently published sika deer genome**

Supplementary Table S6 **Comparison of chromosomes with the recently published sika deer genome**

Supplementary Table S7 **Assessment of the completeness and accuracy of the haplotype-resolved genome of sika deer**

Supplementary Table S8 **Summary of functional annotation in the haplotype-resolved genome of sika deer**

Supplementary Table S9 **Summary of mapping ratio of de novo assembled transcripts (Hap1)**

Supplementary Table S10 **Summary of mapping ratio of de novo assembled transcripts (Hap2)**

Supplementary Table S11 **Overview of predicted ncRNAs in the haplotype-resolved genome of sika deer**

Supplementary Table S12 **Summary of repeat sequence in the haplotype-resolved genome of sika deer**

Supplementary Table S13 **Summary of chromosome information of the haplotype-resolved genome of sika deer**

Supplementary Table S14 **KEGG enrichment analysis of expanded gene families**

Supplementary Table S15 **KEGG enrichment analysis of contracted gene families**

Supplementary Table S16 **Positively selected genes identified**

Supplementary Table S17 **KEGG enrichment analysis of positively selected genes**

Supplementary Table S18 **KEGG enrichment analysis of genes located in the inversion regions of Chr1 in sika deer**

Supplementary Table S19 **KEGG enrichment analysis of genes located in the inversion regions of Chr28 in sika deer**

Supplementary Table S20 **Summary of the distribution of alleles on homologous chromosomes**

Supplementary Table S21 **Summary of structural variation between two haplotypes**
