# Supplementary material for: Haplotype-resolved Genome of Sika Deer Reveals Allele-specific Gene Expression and Chromosome Evolution
Source: Genomics Proteomics Bioinformatics. 2022 Nov 15;21(3):470–82. doi: 10.1016/j.gpb.2022.11.001 (PMC10787017; doi:10.1016/j.gpb.2022.11.001)
Supplement: Supplementary Table S1 — The statistics of PacBio sequencing data [file mmc1.docx]

**Table S1 The statistics of PacBio sequencing data**

| **Cells** | **Read base (Gb)** | **Read number** | **Read length (max)** | **Read length (mean)** | **Read length (N50)** |
| --- | --- | --- | --- | --- | --- |
| PacBio - 01 | 36.47 | 1,804,756 | 54,996 | 20,206 | 20,244 |
| PacBio - 02 | 30.50 | 1,515,862 | 59,424 | 20,122 | 20,166 |
| PacBio - 03 | 29.42 | 1,452,363 | 54,530 | 20,254 | 20,298 |
