# Supplementary material for: Haplotype-resolved Genome of Sika Deer Reveals Allele-specific Gene Expression and Chromosome Evolution
Source: Genomics Proteomics Bioinformatics. 2022 Nov 15;21(3):470–82. doi: 10.1016/j.gpb.2022.11.001 (PMC10787017; doi:10.1016/j.gpb.2022.11.001)
Supplement: Supplementary Table S2 — Summary of the haplotype-resolved genome of sika deer [file mmc2.docx]

**Table S2** **Summary of the haplotype-resolved genome of sika deer**

|  |  | **Scaffolds** | **Contigs** |
| --- | --- | --- | --- |
| Hap1 | Total number (>) | 2370 | 3283 |
|  | Total length of (bp) | 2,711,193,409 | 2,710,736,909 |
|  | Gap number (bp) | 456,500 | 0 |
|  | Average length (bp) | 1,143,963.38 | 825,689 |
|  | N50 length (bp) | 77,599,026 | 34,977,138 |
|  | N90 length (bp) | 43,791,049 | 2,046,827 |
|  | Maximum length (bp) | 149,544,992 | 100,494,534 |
|  | Minimum length (bp) | 5000 | 5000 |
|  | GC content is (%) | 42.06 | 42.06 |
| Hap2 | Total number (>) | 2387 | 3113 |
|  | Total length of (bp) | 2,557,060,714 | 2,556,697,714 |
|  | Gap number (bp) | 363,000 | 0 |
|  | Average length (bp) | 1,071,244.5 | 821,297 |
|  | N50 length (bp) | 77,375,352 | 38,088,857 |
|  | N90 length (bp) | 43,809,624 | 2,930,517 |
|  | Maximum length (bp) | 148,685,516 | 102,248,312 |
|  | Minimum length (bp) | 5000 | 5000 |
|  | GC content is (%) | 42.17 | 42.17 |

*Note*: Hap1, haplotype 1; Hap2, haplotype 2.
