# Supplementary material for: Haplotype-resolved Genome of Sika Deer Reveals Allele-specific Gene Expression and Chromosome Evolution
Source: Genomics Proteomics Bioinformatics. 2022 Nov 15;21(3):470–82. doi: 10.1016/j.gpb.2022.11.001 (PMC10787017; doi:10.1016/j.gpb.2022.11.001)
Supplement: Supplementary Table S3 — Statistics information of chromosome-level of the haplotype-resolved genome [file mmc3.docx]

**Table S3 Statistics information of chromosome-level of the haplotype-resolved genome**

| **ID** | **Length** | **Covered base** | **Total base number** | **Coverage %** | **Mean depth** |
| --- | --- | --- | --- | --- | --- |
| chr1.1 | 149,544,992 | 149,195,032 | 4,940,350,832 | 99.77 | 33.04 |
| chr2.1 | 130,670,556 | 130,611,004 | 4,115,685,706 | 99.95 | 31.50 |
| chr3.1 | 116,586,825 | 116,451,190 | 3,809,676,003 | 99.88 | 32.68 |
| chr4.1 | 114,900,101 | 114,742,503 | 3,793,889,863 | 99.86 | 33.02 |
| chr5.1 | 105,544,026 | 105,521,631 | 3,366,020,167 | 99.98 | 31.89 |
| chr6.1 | 100,494,534 | 100,493,162 | 3,210,160,155 | 100.00 | 31.94 |
| chr7.1 | 98,146,092 | 98,113,895 | 3,186,102,892 | 99.97 | 32.46 |
| chr8.1 | 95,044,469 | 95,043,387 | 3,072,554,264 | 100.00 | 32.33 |
| chr9.1 | 93,262,717 | 93,214,755 | 3,242,879,115 | 99.95 | 34.77 |
| chr10.1 | 86,877,070 | 86,807,119 | 2,723,249,498 | 99.92 | 31.35 |
| chr11.1 | 80,453,856 | 80,387,631 | 2,643,084,404 | 99.92 | 32.85 |
| chr12.1 | 77,599,026 | 77,586,919 | 2,481,101,158 | 99.98 | 31.97 |
| chr13.1 | 77,061,120 | 77,051,725 | 2,640,886,408 | 99.99 | 34.27 |
| chr14.1 | 76,221,915 | 76,100,204 | 2,583,778,616 | 99.84 | 33.90 |
| chr15.1 | 68,598,998 | 68,555,682 | 2,258,407,821 | 99.94 | 32.92 |
| chr16.1 | 67,305,482 | 67,174,851 | 2,120,391,366 | 99.81 | 31.50 |
| chr17.1 | 62,587,973 | 62,577,108 | 2,093,629,811 | 99.98 | 33.45 |
| chr18.1 | 62,421,056 | 62,365,827 | 2,048,785,013 | 99.91 | 32.82 |
| chr19.1 | 61,719,192 | 61,646,603 | 2,079,025,570 | 99.88 | 33.69 |
| chr20.1 | 59,426,514 | 59,422,447 | 1,895,862,593 | 99.99 | 31.90 |
| chr21.1 | 59,690,157 | 59,666,854 | 1,980,627,987 | 99.96 | 33.18 |
| chr22.1 | 59,496,780 | 59,448,623 | 1,940,943,704 | 99.92 | 32.62 |
| chr23.1 | 54,288,043 | 54,287,983 | 1,725,827,380 | 100.00 | 31.79 |
| chr24.1 | 53,687,539 | 53,682,580 | 1,738,199,719 | 99.99 | 32.38 |
| chr25.1 | 53,611,217 | 53,504,237 | 1,733,980,940 | 99.80 | 32.34 |
| chr26.1 | 52,762,115 | 52,702,788 | 1,717,061,534 | 99.89 | 32.54 |
| chr27.1 | 52,098,822 | 51,679,252 | 1,997,204,820 | 99.19 | 38.33 |
| chr28.1 | 48,869,665 | 48,857,940 | 1,740,548,670 | 99.98 | 35.62 |
| chr29.1 | 45,589,179 | 45,410,380 | 1,620,442,809 | 99.61 | 35.54 |
| chr30.1 | 43,791,049 | 43,769,709 | 1,563,692,898 | 99.95 | 35.71 |
| chr31.1 | 41,137,703 | 41,066,357 | 1,427,607,421 | 99.83 | 34.70 |
| chr32.1 | 39,194,247 | 39,157,583 | 1,312,423,008 | 99.91 | 33.49 |
| chrX | 140,315,362 | 139,903,947 | 2,388,805,663 | 99.71 | 17.02 |
| Genome | 2,551,302,524 | 2,548,309,552 | 81,792,244,825 | 99.88 | 32.06 |
| chr1.2 | 148,685,516 | 148,217,242 | 3,142,569,235 | 99.69 | 21.14 |
| chr2.2 | 130,037,195 | 129,962,836 | 2,673,235,132 | 99.94 | 20.56 |
| chr3.2 | 115,738,209 | 115,589,167 | 2,460,796,489 | 99.87 | 21.26 |
| chr4.2 | 114,035,425 | 113,914,914 | 2,448,567,942 | 99.89 | 21.47 |
| chr5.2 | 106,036,145 | 106,004,232 | 2,220,898,466 | 99.97 | 20.94 |
| chr6.2 | 103,921,317 | 103,824,878 | 2,167,483,630 | 99.91 | 20.86 |
| chr7.2 | 99,738,844 | 99,567,603 | 2,129,119,401 | 99.83 | 21.35 |
| chr8.2 | 95,429,253 | 95,379,885 | 2,023,998,903 | 99.95 | 21.21 |
| chr9.2 | 93,148,033 | 93,114,430 | 1,964,091,614 | 99.96 | 21.09 |
| chr10.2 | 86,920,822 | 86,762,175 | 1,800,337,340 | 99.82 | 20.71 |
| chr11.2 | 80,611,781 | 80,423,501 | 1,752,180,854 | 99.77 | 21.74 |
| chr12.2 | 77,602,908 | 77,555,050 | 1,624,368,777 | 99.94 | 20.93 |
| chr13.2 | 76,550,629 | 76,532,582 | 1,600,119,564 | 99.98 | 20.90 |
| chr14.2 | 77,375,352 | 77,073,947 | 1,670,492,942 | 99.61 | 21.59 |
| chr15.2 | 69,021,737 | 68,892,626 | 1,477,512,160 | 99.81 | 21.41 |
| chr16.2 | 67,266,050 | 67,177,580 | 1,392,503,882 | 99.87 | 20.70 |
| chr17.2 | 62,411,649 | 62,328,490 | 1,362,242,706 | 99.87 | 21.83 |
| chr18.2 | 62,278,049 | 62,259,376 | 1,340,985,794 | 99.97 | 21.53 |
| chr19.2 | 62,124,366 | 61,879,046 | 1,344,113,223 | 99.61 | 21.64 |
| chr20.2 | 60,029,666 | 60,008,631 | 1,227,524,516 | 99.96 | 20.45 |
| chr21.2 | 59,492,455 | 59,437,720 | 1,256,249,316 | 99.91 | 21.12 |
| chr22.2 | 59,098,087 | 59,059,916 | 1,247,336,904 | 99.94 | 21.11 |
| chr23.2 | 54,250,704 | 54,247,162 | 1,126,178,963 | 99.99 | 20.76 |
| chr24.2 | 53,645,554 | 53,638,386 | 1,135,221,090 | 99.99 | 21.16 |
| chr25.2 | 53,266,807 | 52,877,441 | 1,085,228,306 | 99.27 | 20.37 |
| chr26.2 | 51,927,931 | 51,926,877 | 1,090,861,503 | 100.00 | 21.01 |
| chr27.2 | 51,229,442 | 50,230,750 | 1,089,614,410 | 98.05 | 21.27 |
| chr28.2 | 48,913,244 | 48,839,352 | 995,062,667 | 99.85 | 20.34 |
| chr29.2 | 46,965,604 | 46,651,133 | 940,919,765 | 99.33 | 20.03 |
| chr30.2 | 43,809,624 | 43,771,019 | 915,566,192 | 99.91 | 20.90 |
| chr31.2 | 41,744,783 | 41,594,112 | 839,392,533 | 99.64 | 20.11 |
| chr32.2 | 39,154,015 | 39,048,305 | 810,463,021 | 99.73 | 20.70 |
| chrY | 22,304,132 | 22,078,337 | 392,834,694 | 98.99 | 17.61 |
| Genome | 2,557,060,714 | 2,505,015,925 | 52,556,897,188 | 97.96 | 20.55 |
