# Supplementary material for: Haplotype-resolved Genome of Sika Deer Reveals Allele-specific Gene Expression and Chromosome Evolution
Source: Genomics Proteomics Bioinformatics. 2022 Nov 15;21(3):470–82. doi: 10.1016/j.gpb.2022.11.001 (PMC10787017; doi:10.1016/j.gpb.2022.11.001)
Supplement: Supplementary Table S4 — Summary of BUSCOs recovered in the haplotype-resolved genome of sika deer [file mmc4.docx]

**Table S4 Summary of BUSCOs recovered in the haplotype-resolved genome of sika deer**

| **Type** | **Haplotype 1** | | **Haplotype 2** | |
| --- | --- | --- | --- | --- |
|  | **Number** | **Percentage (%)** | **Number** | **Percentage (%)** |
| Complete BUSCOs (C) | 3879 | 94.5 | 3897 | 95.0 |
| Complete and single-copy BUSCOs (S) | 3805 | 92.7 | 3814 | 93.0 |
| Complete and duplicated BUSCOs (D) | 74 | 1.8 | 83 | 2.0 |
| Fragmented BUSCOs (F) | 78 | 1.9 | 75 | 1.8 |
| Missing BUSCOs (M) | 147 | 3.6 | 132 | 3.2 |
| BUSCO assessment |  | 94.5 |  | 95.0 |
| Total | 4104 |  | 4104 |  |

*Note*: BUSCO, Benchmarking Universal Single-Copy Orthologs.
