# Supplementary material for: Haplotype-resolved Genome of Sika Deer Reveals Allele-specific Gene Expression and Chromosome Evolution
Source: Genomics Proteomics Bioinformatics. 2022 Nov 15;21(3):470–82. doi: 10.1016/j.gpb.2022.11.001 (PMC10787017; doi:10.1016/j.gpb.2022.11.001)
Supplement: Supplementary Table S5 — Summary of comparison with the recently published sika deer genome [file mmc5.docx]

**Table S5**  **Summary of comparison with the recently published sika deer genome**

| **Species** | **Haplotype** | **Gender** | **Chromosome** | **Contig N50 (bp)** | **Complete BUSCOs** | **Repetitive sequences** | **Gene number** | **Functional annotation** |
| --- | --- | --- | --- | --- | --- | --- | --- | --- |
| *Cervus nippon* | — | Female | 32 autosomes+ X | 23,559,432 | 94.6% | 45.38% | 21,449 | 90.1% |
| *Cervus nippon*  (In the present study) | Hap1 | Male | 32 autosomes+ X | 34,977,138 | 94.5% | 42.39% | 22,144 | 93.8% |
|  | Hap2 |  | 32 autosomes+ Y | 38,088,857 | 95.0% | 43.06% | 18,705 | 91.6% |
