# Supplementary material for: Haplotype-resolved Genome of Sika Deer Reveals Allele-specific Gene Expression and Chromosome Evolution
Source: Genomics Proteomics Bioinformatics. 2022 Nov 15;21(3):470–82. doi: 10.1016/j.gpb.2022.11.001 (PMC10787017; doi:10.1016/j.gpb.2022.11.001)
Supplement: Supplementary Table S6 — Comparison of chromosomes with the recently published sika deer genome [file mmc6.docx]

**Table S6 Comparison of chromosomes with the recently published sika deer genome**

| **Haplotype-resolved genome**  **(Present study)** | **Sika deer genome (Xing et al)** |
| --- | --- |
| chr1.1 | chr1 |
| chr2.1 | chr2 |
| chr3.1 | chr3 |
| chr4.1 | chr4 |
| chr5.1 | chr5 |
| chr6.1 | chr6 |
| chr7.1 | chr7 |
| chr8.1 | chr8 |
| chr9.1 | chr9 |
| chr10.1 | chr10 |
| chr11.1 | chr11 |
| chr12.1 | chr12 |
| chr13.1 | chr13 |
| chr14.1 | chr14 |
| chr15.1 | chr15 |
| chr16.1 | chr16 |
| chr17.1 | chr17 |
| chr18.1 | chr18 |
| chr19.1 | chr26 |
| chr20.1 | chr20 |
| chr21.1 | chr19 |
| chr22.1 | chr21 |
| chr23.1 | chr22 |
| chr24.1 | chr23 |
| chr25.1 | chr25 |
| chr26.1 | chr24 |
| chr27.1 | chr27 |
| chr28.1 | chr28 |
| chr29.1 | chr29 |
| chr30.1 | chr30 |
| chr31.1 | chr31 |
| chr32.1 | chr32 |
| chrX | chrX |
| chrY | — |
