# Supplementary material for: Haplotype-resolved Genome of Sika Deer Reveals Allele-specific Gene Expression and Chromosome Evolution
Source: Genomics Proteomics Bioinformatics. 2022 Nov 15;21(3):470–82. doi: 10.1016/j.gpb.2022.11.001 (PMC10787017; doi:10.1016/j.gpb.2022.11.001)
Supplement: Supplementary Table S7 — Assessment of the completeness and accuracy of the haplotype-resolved genome of sika deer [file mmc7.docx]

**Table S7**  **Assessment of the completeness and accuracy of the haplotype-resolved genome of sika deer**

| **Species** | **Method** | **Haplotype** | **Mapping ratio** |
| --- | --- | --- | --- |
| Sika deer  (Haplotype-resolved) | CEGMA | Hap1 | 99.57% |
|  |  | Hap2 | 98.28% |
|  | Illumina reads (coverage) | Hap1 | 99.88% |
|  |  | Hap2 | 97.96% |
|  | Illumina reads (mapping rate) | Hap1 | 99.83% |
|  |  | Hap2 | 98.92% |
|  | EST | Hap1 | 98.32% |
|  |  | Hap2 | 95.81% |

*Note*: CEGMA, Core Eukaryotic Genes Mapping Approach; EST, expressed sequence tag.
