# Supplementary material for: Haplotype-resolved Genome of Sika Deer Reveals Allele-specific Gene Expression and Chromosome Evolution
Source: Genomics Proteomics Bioinformatics. 2022 Nov 15;21(3):470–82. doi: 10.1016/j.gpb.2022.11.001 (PMC10787017; doi:10.1016/j.gpb.2022.11.001)
Supplement: Supplementary Table S8 — Summary of functional annotation in the haplotype-resolved genome of sika deer [file mmc8.docx]

**Table S8 Summary of functional annotation in the haplotype-resolved genome of sika deer**

|  |  | **Total** | **Nr** | **Swiss-Prot** | **KEGG** | **KOG** | **TrEMBL** | **Interpro** | **GO** | **Overall** |
| --- | --- | --- | --- | --- | --- | --- | --- | --- | --- | --- |
| Hap1 | Number | 22,144 | 20,569 | 18,862 | 17,709 | 15,903 | 20,089 | 20,233 | 13,810 | 20,771 |
|  | Percentage | 100% | 92.89% | 85.18% | 79.97% | 71.82% | 90.72% | 91.37% | 62.36% | 93.80% |
| Hap2 | Number | 18,705 | 17,041 | 16,602 | 15,411 | 14,202 | 16,971 | 16,634 | 12,420 | 17,133 |
|  | Percentage | 100% | 91.10% | 88.76% | 82.39% | 75.93% | 90.73% | 88.93% | 66.40% | 91.60% |

*Note*: Nr, Non-Redundant Protein Sequence Database; Swiss-Prot; KEGG, Kyoto encyclopedia of genes and genomes; KOG, Clusters of orthologous groups for eukaryotic complete genomes; TrEMBL, Translation of EMB; Interpro; GO, Gene Ontology.
