# Supplementary material for: Haplotype-resolved Genome of Sika Deer Reveals Allele-specific Gene Expression and Chromosome Evolution
Source: Genomics Proteomics Bioinformatics. 2022 Nov 15;21(3):470–82. doi: 10.1016/j.gpb.2022.11.001 (PMC10787017; doi:10.1016/j.gpb.2022.11.001)
Supplement: Supplementary Table S10 — Summary of mapping ratio of de novo assembled transcripts (Hap2) [file mmc10.docx]

**Table S10 Summary of mapping ratio of *de novo* assembled transcripts (Hap2)**

| **ID** | **Samples** | **Mapping ratio** |
| --- | --- | --- |
| [SRR8002919](https://trace.ncbi.nlm.nih.gov/Traces/sra/?run=SRR8002919) | Testis | 92.22% |
| [SRR8002928](https://trace.ncbi.nlm.nih.gov/Traces/sra/?run=SRR8002928) | Antler_velvet | 94.21% |
| [SRR8002942](https://trace.ncbi.nlm.nih.gov/Traces/sra/?run=SRR8002942) | Spleen | 92.25% |
| [SRR8002943](https://trace.ncbi.nlm.nih.gov/Traces/sra/?run=SRR8002943) | Muscle | 95.11% |
| [SRR8002944](https://trace.ncbi.nlm.nih.gov/Traces/sra/?run=SRR8002944) | Lung | 93.96% |
| [SRR8002945](https://trace.ncbi.nlm.nih.gov/Traces/sra/?run=SRR8002945) | Kidney | 94.27% |
| [SRR8002957](https://trace.ncbi.nlm.nih.gov/Traces/sra/?run=SRR8002957) | Heart | 94.73% |
| [SRR8002962](https://trace.ncbi.nlm.nih.gov/Traces/sra/?run=SRR8002962) | Liver | 94.35% |
| SRR9618241 | Antler-EP-1 | 96.59% |
| SRR9618242 | Antler-EP-2 | 96.54% |
| SRR9618239 | Antler-EP-3 | 96.72% |
| SRR9618240 | Antler-MP-1 | 96.69% |
| SRR9618237 | Antler-MP-2 | 96.67% |
| SRR9618238 | Antler-MP-3 | 96.82% |
| SRR9618235 | Antler-LP-1 | 96.63% |
| SRR9618236 | Antler-LP-2 | 97.04% |
| SRR9618243 | Antler-LP-3 | 96.95% |
