# Supplementary material for: Haplotype-resolved Genome of Sika Deer Reveals Allele-specific Gene Expression and Chromosome Evolution
Source: Genomics Proteomics Bioinformatics. 2022 Nov 15;21(3):470–82. doi: 10.1016/j.gpb.2022.11.001 (PMC10787017; doi:10.1016/j.gpb.2022.11.001)
Supplement: Supplementary Table S11 — Overview of predicted ncRNAs in the haplotype-resolved genome of sika deer [file mmc11.docx]

**Table S11 Overview of predicted ncRNAs in the haplotype-resolved genome of sika deer**

|  | **Type** |  | **Copy (w)** | **Average length (bp)** | **Total length (bp)** | **Percentage of genome (%)** |
| --- | --- | --- | --- | --- | --- | --- |
| Hap1 | miRNA |  | 883 | 79.82332956 | 70,484 | 0.0026 |
|  | tRNA |  | 267,037 | 73.29876384 | 19,573,482 | 0.721951 |
|  | rRNA | rRNA | 8698 | 96.4818349 | 839,199 | 0.030953 |
|  |  | 18S | 248 | 230.9193548 | 57,268 | 0.002112 |
|  |  | 28S | 561 | 182.6131907 | 102,446 | 0.003779 |
|  |  | 5.8S | 14 | 128.6428571 | 1801 | 0.000066 |
|  |  | 5S | 7875 | 86.05511111 | 677,684 | 0.024996 |
|  | snRNA | snRNA | 1965 | 114.3109415 | 224,621 | 0.008285 |
|  |  | CD-box | 287 | 92.73519164 | 26,615 | 0.000982 |
|  |  | HACA-box | 281 | 136.2135231 | 38,276 | 0.001412 |
|  |  | splicing | 1366 | 113.2371889 | 154,682 | 0.005705 |
| Hap2 | miRNA |  | 815 | 79.91042945 | 65,127 | 0.002547 |
|  | tRNA |  | 253,977 | 73.3057206 | 18,617,967 | 0.7281 |
|  | rRNA | rRNA | 9016 | 111.7830524 | 1,007,836 | 0.039414 |
|  |  | 18S | 324 | 352.25 | 114,129 | 0.004463 |
|  |  | 28S | 905 | 247.0232044 | 223,556 | 0.008743 |
|  |  | 5.8S | 40 | 139.65 | 5586 | 0.000218 |
|  |  | 5S | 7747 | 85.78352911 | 664,565 | 0.025989 |
|  | snRNA | snRNA | 1967 | 115.3202847 | 226,835 | 0.008871 |
|  |  | CD-box | 267 | 93.06741573 | 24,849 | 0.000972 |
|  |  | HACA-box | 300 | 135.1533333 | 40,546 | 0.001586 |
|  |  | splicing | 1364 | 114.2844575 | 155,884 | 0.006096 |

*Note*: miRNA, microRNA; tRNA, transfer RNA; rRNA, ribosomal RNA; snRNA, small nuclear RNA.
