# Supplementary material for: Haplotype-resolved Genome of Sika Deer Reveals Allele-specific Gene Expression and Chromosome Evolution
Source: Genomics Proteomics Bioinformatics. 2022 Nov 15;21(3):470–82. doi: 10.1016/j.gpb.2022.11.001 (PMC10787017; doi:10.1016/j.gpb.2022.11.001)
Supplement: Supplementary Table S12 — Summary of repeat sequence in the haplotype-resolved genome of sika deer [file mmc12.docx]

**Table S12** **Summary of repeat sequence in the haplotype-resolved genome of sika deer**

|  | **Type** | **Repeat size** | **Ratio (%)** |
| --- | --- | --- | --- |
| Hap1 | Trf | 102,717,282 | 3.79 |
|  | Repeatmasker | 843,751,130 | 31.12 |
|  | Proteinmask | 399,361,526 | 14.73 |
|  | *De novo* | 1,001,940,071 | 36.96 |
|  | Total | 1,149,328,479 | 42.39 |
| Hap2 | Trf | 102,587,977 | 4.01 |
|  | Repeatmasker | 783,979,888 | 30.66 |
|  | Proteinmask | 399,220,874 | 15.61 |
|  | *De novo* | 1,001,944,660 | 39.18 |
|  | Total | 1,101,048,944 | 43.06 |
