# Supplementary material for: Haplotype-resolved Genome of Sika Deer Reveals Allele-specific Gene Expression and Chromosome Evolution
Source: Genomics Proteomics Bioinformatics. 2022 Nov 15;21(3):470–82. doi: 10.1016/j.gpb.2022.11.001 (PMC10787017; doi:10.1016/j.gpb.2022.11.001)
Supplement: Supplementary Table S13 — Summary of chromosome information of the haplotype-resolved genome of sika deer [file mmc13.docx]

**Table S13 Summary of chromosome information of the haplotype-resolved genome of sika deer**

|  | **Gene number** | **Average mRNA length of gene** | **Average exon number of gene** | **Average intron number of gene** |
| --- | --- | --- | --- | --- |
| chr1.1 | 1959 | 1273 | 9 | 8 |
| chr1.2 | 1718 | 1172 | 8 | 7 |
| chr2.1 | 1679 | 1444 | 10 | 9 |
| chr2.2 | 1469 | 1340 | 9 | 8 |
| chr3.1 | 1212 | 1262 | 9 | 8 |
| chr3.2 | 1072 | 1197 | 8 | 7 |
| chr4.1 | 781 | 1231 | 8 | 7 |
| chr4.2 | 648 | 1191 | 8 | 7 |
| chr5.1 | 1082 | 1383 | 9 | 8 |
| chr5.2 | 958 | 1318 | 8 | 7 |
| chr6.1 | 814 | 1402 | 10 | 9 |
| chr6.2 | 785 | 1291 | 9 | 8 |
| chr7.1 | 889 | 1323 | 9 | 8 |
| chr7.2 | 811 | 1218 | 9 | 8 |
| chr8.1 | 667 | 1215 | 9 | 8 |
| chr8.2 | 606 | 1183 | 9 | 8 |
| chr9.1 | 740 | 1304 | 9 | 8 |
| chr9.2 | 654 | 1226 | 9 | 8 |
| chr10.1 | 462 | 1180 | 8 | 7 |
| chr10.2 | 414 | 1102 | 8 | 7 |
| chr11.1 | 738 | 1236 | 8 | 7 |
| chr11.2 | 695 | 1168 | 7 | 6 |
| chr12.1 | 481 | 1270 | 8 | 7 |
| chr12.2 | 405 | 1176 | 8 | 7 |
| chr13.1 | 640 | 1385 | 10 | 9 |
| chr13.2 | 556 | 1303 | 9 | 8 |
| chr14.1 | 378 | 1399 | 10 | 9 |
| chr14.2 | 340 | 1257 | 9 | 8 |
| chr15.1 | 349 | 1270 | 9 | 8 |
| chr15.2 | 292 | 1228 | 9 | 8 |
| chr16.1 | 579 | 1259 | 8 | 7 |
| chr16.2 | 535 | 1107 | 7 | 6 |
| chr17.1 | 231 | 1194 | 9 | 8 |
| chr17.2 | 223 | 1136 | 8 | 7 |
| chr18.1 | 380 | 1221 | 8 | 7 |
| chr18.2 | 356 | 1140 | 7 | 6 |
| chr19.1 | 277 | 1175 | 8 | 7 |
| chr19.2 | 273 | 1100 | 8 | 7 |
| chr20.1 | 643 | 1294 | 9 | 8 |
| chr20.2 | 582 | 1261 | 9 | 8 |
| chr21.1 | 556 | 1483 | 10 | 9 |
| chr21.2 | 530 | 1354 | 9 | 8 |
| chr22.1 | 359 | 1219 | 8 | 7 |
| chr22.2 | 330 | 1101 | 7 | 6 |
| chr23.1 | 574 | 1375 | 10 | 9 |
| chr23.2 | 461 | 1304 | 9 | 8 |
| chr24.1 | 466 | 1338 | 10 | 9 |
| chr24.2 | 394 | 1419 | 10 | 9 |
| chr25.1 | 675 | 1184 | 8 | 7 |
| chr25.2 | 607 | 1109 | 7 | 6 |
| chr26.1 | 191 | 1029 | 7 | 6 |
| chr26.2 | 175 | 976 | 7 | 6 |
| chr27.1 | 338 | 1261 | 9 | 8 |
| chr27.2 | 284 | 1245 | 9 | 8 |
| chr28.1 | 551 | 1325 | 9 | 8 |
| chr28.2 | 499 | 1212 | 8 | 7 |
| chr29.1 | 369 | 1227 | 9 | 8 |
| chr29.2 | 352 | 1114 | 8 | 7 |
| chr30.1 | 257 | 1147 | 8 | 7 |
| chr30.2 | 232 | 1073 | 7 | 6 |
| chr31.1 | 681 | 1425 | 10 | 9 |
| chr31.2 | 621 | 1278 | 9 | 8 |
| chr32.1 | 290 | 1212 | 8 | 7 |
| chr32.2 | 255 | 1094 | 7 | 6 |
| chrX | 970 | 1008 | 7 | 6 |
| chrY | 111 | 883 | 6 | 5 |

*Note*: CDS, coding sequence.
