# Supplementary material for: Haplotype-resolved Genome of Sika Deer Reveals Allele-specific Gene Expression and Chromosome Evolution
Source: Genomics Proteomics Bioinformatics. 2022 Nov 15;21(3):470–82. doi: 10.1016/j.gpb.2022.11.001 (PMC10787017; doi:10.1016/j.gpb.2022.11.001)
Supplement: Supplementary Table S14 — KEGG enrichment analysis of expanded gene families [file mmc14.docx]

**Table S14** **KEGG enrichment analysis of expanded gene families**

| **Pathway ID** | **Pathway** | **Gene number** | ***P* value** |
| --- | --- | --- | --- |
| ko05322 | Systemic lupus erythematosus | 98 | 7.755E−42 |
| ko05150 | Staphylococcus aureus infection | 74 | 1.767E−28 |
| ko05320 | Autoimmune thyroid disease | 65 | 2.495E−28 |
| ko05330 | Allograft rejection | 57 | 3.563E−23 |
| ko05203 | Viral carcinogenesis | 86 | 4.551E−22 |
| ko05416 | Viral myocarditis | 64 | 1.995E−21 |
| ko05332 | Graft-versus-host disease | 47 | 4.227E−19 |
| ko05216 | Thyroid cancer | 42 | 6.673E−19 |
| ko05146 | Amoebiasis | 63 | 1.767E−17 |
| ko04940 | Type I diabetes mellitus | 47 | 2.105E−16 |
| ko05230 | Central carbon metabolism in cancer | 44 | 3.342E−16 |
| ko05169 | Epstein-Barr virus infection | 77 | 1.042E−15 |
| ko05310 | Asthma | 40 | 1.464E−15 |
| ko05170 | Human immunodeficiency virus 1 infection | 74 | 1.862E−14 |
| ko04612 | Antigen processing and presentation | 51 | 4.022E−14 |
| ko05160 | Hepatitis C | 59 | 4.488E−14 |
| ko04672 | Intestinal immune network for IgA production | 40 | 8.82E−14 |
| ko04145 | Phagosome | 61 | 1.951E−13 |
| ko04390 | Hippo signaling pathway | 67 | 3.544E−13 |
| ko05034 | Alcoholism | 55 | 4.329E−13 |
| ko04622 | RIG-I-like receptor signaling pathway | 34 | 7.062E−12 |
| ko04621 | NOD-like receptor signaling pathway | 55 | 1.054E−11 |
| ko05202 | Transcriptional misregulation in cancer | 66 | 3.733E−11 |
| ko04650 | Natural killer cell mediated cytotoxicity | 40 | 1.946E−10 |
| ko05323 | Rheumatoid arthritis | 44 | 3.543E−10 |
| ko04151 | PI3K-Akt signaling pathway | 87 | 1.937E−09 |
| ko05162 | Measles | 47 | 2.08E−09 |
| ko05144 | Malaria | 31 | 3.614E−09 |
| ko04064 | NF-kappa B signaling pathway | 43 | 5.634E−09 |
| ko05205 | Proteoglycans in cancer | 62 | 5.917E−09 |
| ko05161 | Hepatitis B | 50 | 6.447E−09 |
| ko05142 | Chagas disease (American trypanosomiasis) | 43 | 7.571E−09 |
| ko04114 | Oocyte meiosis | 43 | 8.761E−09 |
| ko04514 | Cell adhesion molecules (CAMs) | 52 | 9.106E−09 |
| ko05165 | Human papillomavirus infection | 81 | 1.619E−08 |
| ko04212 | Longevity regulating pathway - worm | 35 | 3.823E−08 |
| ko05321 | Inflammatory bowel disease (IBD) | 33 | 5.092E−08 |
| ko04391 | Hippo signaling pathway - fly | 40 | 1.158E−07 |
| ko04110 | Cell cycle | 41 | 1.323E−07 |
| ko05217 | Basal cell carcinoma | 25 | 6.639E−07 |
| ko05130 | Pathogenic Escherichia coli infection | 53 | 7.039E−07 |
| ko05340 | Primary immunodeficiency | 21 | 7.069E−07 |
| ko00590 | Arachidonic acid metabolism | 27 | 1.697E−06 |
| ko04013 | MAPK signaling pathway - fly | 36 | 3.6E−06 |
| ko04660 | T cell receptor signaling pathway | 33 | 1.284E−05 |
| ko04658 | Th1 and Th2 cell differentiation | 33 | 1.284E−05 |
| ko05166 | Human T-cell leukemia virus 1 infection | 50 | 2.062E−05 |
| ko05204 | Chemical carcinogenesis | 22 | 2.308E−05 |
| ko04666 | Fc gamma R-mediated phagocytosis | 26 | 2.544E−05 |
| ko05152 | Tuberculosis | 39 | 3.114E−05 |
| ko05167 | Kaposi sarcoma-associated herpesvirus infection | 38 | 4.466E−05 |
| ko04933 | AGE-RAGE signaling pathway in diabetic complications | 27 | 6.592E−05 |
| ko05200 | Pathways in cancer | 89 | 7.591E−05 |
| ko05235 | PD-L1 expression and PD-1 checkpoint pathway in cancer | 28 | 7.845E−05 |
| ko04020 | Calcium signaling pathway | 47 | 0.000109 |
| ko04974 | Protein digestion and absorption | 29 | 0.0001124 |
| ko04630 | Jak-STAT signaling pathway | 33 | 0.0001317 |
| ko05143 | African trypanosomiasis | 15 | 0.0001659 |
| ko04726 | Serotonergic synapse | 25 | 0.0001995 |
| ko05135 | Yersinia infection | 36 | 0.0002303 |
| ko00730 | Thiamine metabolism | 8 | 0.0002318 |
| ko01523 | Antifolate resistance | 19 | 0.0002779 |
| ko04659 | Th17 cell differentiation | 30 | 0.000297 |
| ko04916 | Melanogenesis | 26 | 0.0003467 |
| ko00140 | Steroid hormone biosynthesis | 20 | 0.0003528 |
| ko04530 | Tight junction | 43 | 0.0003918 |
| ko05226 | Gastric cancer | 34 | 0.0004223 |
| ko00310 | Lysine degradation | 22 | 0.0004569 |
| ko05206 | MicroRNAs in cancer | 36 | 0.0005343 |
| ko04662 | B cell receptor signaling pathway | 21 | 0.0006724 |
| ko00790 | Folate biosynthesis | 12 | 0.0011028 |
| ko04934 | Cushing syndrome | 31 | 0.0016711 |
| ko04976 | Bile secretion | 23 | 0.0019921 |
| ko00513 | Various types of N-glycan biosynthesis | 14 | 0.0023694 |
| ko04623 | Cytosolic DNA-sensing pathway | 14 | 0.0023694 |
| ko02010 | ABC transporters | 19 | 0.0029221 |
| ko04217 | Necroptosis | 33 | 0.0031212 |
| ko00830 | Retinol metabolism | 17 | 0.0034727 |
| ko00591 | Linoleic acid metabolism | 9 | 0.0037901 |
| ko04926 | Relaxin signaling pathway | 27 | 0.0040388 |
| ko04970 | Salivary secretion | 20 | 0.0053352 |
| ko00510 | N-Glycan biosynthesis | 14 | 0.0066447 |
| ko04060 | Cytokine-cytokine receptor interaction | 37 | 0.0069767 |
| ko04611 | Platelet activation | 28 | 0.0072858 |
| ko05132 | Salmonella infection | 19 | 0.0103385 |
| ko04550 | Signaling pathways regulating pluripotency of stem cells | 25 | 0.0118641 |
| ko04150 | mTOR signaling pathway | 29 | 0.0121044 |
| ko04540 | Gap junction | 19 | 0.0138321 |
| ko04640 | Hematopoietic cell lineage | 19 | 0.0148363 |
| ko05140 | Leishmaniasis | 16 | 0.017136 |
| ko05224 | Breast cancer | 26 | 0.0227599 |
| ko04360 | Axon guidance | 32 | 0.0304435 |
| ko04137 | Mitophagy - animal | 13 | 0.0387532 |
| ko05414 | Dilated cardiomyopathy (DCM) | 24 | 0.0391219 |
| ko05168 | Herpes simplex virus 1 infection | 49 | 0.0399119 |
| ko05163 | Human cytomegalovirus infection | 33 | 0.0420617 |
| ko04512 | ECM-receptor interaction | 21 | 0.0424359 |
| ko05225 | Hepatocellular carcinoma | 28 | 0.0429677 |
| ko04210 | Apoptosis | 24 | 0.0470454 |
