# Supplementary material for: Haplotype-resolved Genome of Sika Deer Reveals Allele-specific Gene Expression and Chromosome Evolution
Source: Genomics Proteomics Bioinformatics. 2022 Nov 15;21(3):470–82. doi: 10.1016/j.gpb.2022.11.001 (PMC10787017; doi:10.1016/j.gpb.2022.11.001)
Supplement: Supplementary Table S15 — KEGG enrichment analysis of contracted gene families [file mmc15.docx]

**Table S15 KEGG enrichment analysis of contracted gene families**

| **Pathway ID** | **Pathway** | **Gene number** | ***P* value** |
| --- | --- | --- | --- |
| ko05165 | Human papillomavirus infection | 9 | 0.000323 |
| ko02010 | ABC transporters | 3 | 0.012916 |
| ko00640 | Propanoate metabolism | 2 | 0.013402 |
| ko00620 | Pyruvate metabolism | 2 | 0.015817 |
| ko05222 | Small cell lung cancer | 3 | 0.015934 |
| ko04928 | Parathyroid hormone synthesis, secretion and action | 3 | 0.018263 |
| ko05145 | Toxoplasmosis | 3 | 0.022287 |
| ko04340 | Hedgehog signaling pathway | 2 | 0.025584 |
| ko05143 | African trypanosomiasis | 2 | 0.029534 |
| ko00520 | Amino sugar and nucleotide sugar metabolism | 2 | 0.029534 |
| ko04979 | Cholesterol metabolism | 2 | 0.030351 |
| ko04512 | ECM-receptor interaction | 3 | 0.035521 |
| ko04530 | Tight junction | 4 | 0.040111 |
| ko04510 | Focal adhesion | 4 | 0.040495 |
| ko04390 | Hippo signaling pathway | 4 | 0.043643 |
| ko01212 | Fatty acid metabolism | 2 | 0.046521 |
| ko05418 | Fluid shear stress and atherosclerosis | 3 | 0.047296 |
