# Supplementary material for: Haplotype-resolved Genome of Sika Deer Reveals Allele-specific Gene Expression and Chromosome Evolution
Source: Genomics Proteomics Bioinformatics. 2022 Nov 15;21(3):470–82. doi: 10.1016/j.gpb.2022.11.001 (PMC10787017; doi:10.1016/j.gpb.2022.11.001)
Supplement: Supplementary Table S16 — Positively selected genes identified [file mmc16.docx]

**Table S16 Positively selected genes identified**

| **Gene name** | **Description** |
| --- | --- |
| RBM45 | RNA Binding Motif Protein 45 |
| TUBGCP4 | gamma-tubulin complex component 4 isoform X1 |
| WDR43 | WD repeat-containing protein 43 |
| DNAJC16 | DnaJ Heat Shock Protein Family (Hsp40) Member C16 |
| TMEM17 | transmembrane protein 17 |
| Tmem161b | T161B protein, partial |
| ASPM | PREDICTED: abnormal spindle-like microcephaly-associated protein |
| WDR3 | WD Repeat Domain 3 |
| NFIL3 | Nuclear Factor, Interleukin 3 Regulated |
| PPP4R2 | protein phosphatase 4 regulatory subunit 2 |
| OX=9913 | hypothetical protein FD754_006889 |
| IRAK4 | interleukin-1 receptor-associated kinase 4 |
| CCDC186 | coiled-coil domain-containing protein 186 |
| FAM71D | FAM71D |
| Ttc8 | Tetratricopeptide Repeat Domain 8 |
| WNT8A | WNT8A |
| Yipf3 | Yip1 Domain Family Member 3 |
| HPS6 | HPS6 Biogenesis Of Lysosomal Organelles Complex 2 Subunit 3 |
| BMP4 | bone morphogenetic protein 4 |
| Med19 | PREDICTED: mediator of RNA polymerase II transcription subunit 19 |
| YARS1 | Tyrosyl-TRNA Synthetase 1 |
| NBR1 | NBR1 Autophagy Cargo Receptor |
| TP53 | Tumor Protein P53 |
| CCDC82 | Coiled-Coil Domain Containing 82 |
| MMRN2 | Multimerin 2 |
| PRKCSH | Protein Kinase C Substrate 80K-H |
| GDF9 | Growth Differentiation Factor 9 |
| VEZF1 | vascular endothelial zinc finger 1 |
| GINM1 | glycoprotein integral membrane protein 1 |
| WNT9B | Wnt Family Member 9B |
| KIF20A | Kinesin Family Member 20A |
| TCF24 | Transcription Factor 24 |
| RSAD2 | Radical S-Adenosyl Methionine Domain Containing 2 |
| PSMD12 | Proteasome 26S Subunit, Non-ATPase 12 |
| SMDT1 | Single-Pass Membrane Protein With Aspartate Rich Tail 1 |
| TMEM41A | Transmembrane Protein 41A |
| SLC10A2 | Solute Carrier Family 10 Member 2 |
| SCLY | Selenocysteine Lyase |
| EIF4E | eukaryotic translation initiation factor 4E-like |
| NPY | pro-neuropeptide Y |
| ANKS4B | Ankyrin Repeat And Sterile Alpha Motif Domain Containing 4B |
| LACTB | Lactamase Beta |
