# Supplementary material for: Haplotype-resolved Genome of Sika Deer Reveals Allele-specific Gene Expression and Chromosome Evolution
Source: Genomics Proteomics Bioinformatics. 2022 Nov 15;21(3):470–82. doi: 10.1016/j.gpb.2022.11.001 (PMC10787017; doi:10.1016/j.gpb.2022.11.001)
Supplement: Supplementary Table S17 — KEGG enrichment analysis of positively selected genes [file mmc17.docx]

**Table S17 KEGG enrichment analysis of positively selected genes**

| **Pathway ID** | **Pathway** | **Gene number** | ***P* value** |
| --- | --- | --- | --- |
| ko05217 | Basal cell carcinoma | 4 | 6.597E−05 |
| ko04550 | Signaling pathways regulating pluripotency of stem cells | 3 | 0.00745738 |
| ko05224 | Breast cancer | 3 | 0.00993816 |
| ko05162 | Measles | 3 | 0.01201096 |
| ko05226 | Gastric cancer | 3 | 0.01214816 |
| ko04150 | mTOR signaling pathway | 3 | 0.01242528 |
| ko04310 | Wnt signaling pathway | 3 | 0.01477365 |
| ko05225 | Hepatocellular carcinoma | 3 | 0.01477365 |
| ko04137 | Mitophagy - animal | 2 | 0.01651012 |
| ko03008 | Ribosome biogenesis in eukaryotes | 2 | 0.01882494 |
| ko04211 | Longevity regulating pathway | 2 | 0.0285199 |
| ko04390 | Hippo signaling pathway | 3 | 0.02918186 |
| ko05145 | Toxoplasmosis | 2 | 0.03706509 |
| ko00450 | Selenocompound metabolism | 1 | 0.0378542 |
| ko05205 | Proteoglycans in cancer | 3 | 0.03789485 |
| ko05169 | Epstein-Barr virus infection | 3 | 0.03841414 |
| ko04010 | MAPK signaling pathway | 3 | 0.04052806 |
| ko04916 | Melanogenesis | 2 | 0.04260747 |
| ko04919 | Thyroid hormone signaling pathway | 2 | 0.0435599 |
| ko04722 | Neurotrophin signaling pathway | 2 | 0.0435599 |
| ko00603 | Glycosphingolipid biosynthesis - globo and isoglobo series | 1 | 0.0437023 |
