# Supplementary material for: Haplotype-resolved Genome of Sika Deer Reveals Allele-specific Gene Expression and Chromosome Evolution
Source: Genomics Proteomics Bioinformatics. 2022 Nov 15;21(3):470–82. doi: 10.1016/j.gpb.2022.11.001 (PMC10787017; doi:10.1016/j.gpb.2022.11.001)
Supplement: Supplementary Table S18 — KEGG enrichment analysis of genes located in the inversion regions of Chr1 in sika deer [file mmc18.docx]

**Table S18** **KEGG enrichment analysis of genes located in the inversion regions of Chr1 in sika deer**

| **Pathway ID** | **Pathways** | **Gene number** | ***P* value** |
| --- | --- | --- | --- |
| ko00790 | Folate biosynthesis | 18 | 2.597E−20 |
| ko04913 | Ovarian steroidogenesis | 18 | 1.033E−16 |
| ko05168 | Herpes simplex virus 1 infection | 15 | 1.065E−14 |
| ko00140 | Steroid hormone biosynthesis | 18 | 1.48E−14 |
| ko00590 | Arachidonic acid metabolism | 18 | 1.912E−13 |
| ko01100 | Metabolic pathways | 47 | 0.0005852 |
| ko04146 | Peroxisome | 5 | 0.0160908 |
| ko00640 | Propanoate metabolism | 3 | 0.0229811 |
| ko00051 | Fructose and mannose metabolism | 3 | 0.0229811 |
| ko04122 | Sulfur relay system | 1 | 0.024073 |
| ko00410 | beta-Alanine metabolism | 3 | 0.0258169 |
| ko00630 | Glyoxylate and dicarboxylate metabolism | 3 | 0.0273021 |
| ko00533 | Glycosaminoglycan biosynthesis - keratan sulfate | 1 | 0.02803 |
| ko01200 | Carbon metabolism | 6 | 0.0306448 |
| ko04530 | Tight junction | 9 | 0.0361648 |
| ko00120 | Primary bile acid biosynthesis | 2 | 0.0368717 |
| ko04270 | Vascular smooth muscle contraction | 6 | 0.0397813 |
| ko04970 | Salivary secretion | 5 | 0.0433502 |
| ko05412 | Arrhythmogenic right ventricular cardiomyopathy (ARVC) | 5 | 0.0456299 |
| ko00900 | Terpenoid backbone biosynthesis | 2 | 0.0466107 |
