# Supplementary material for: Haplotype-resolved Genome of Sika Deer Reveals Allele-specific Gene Expression and Chromosome Evolution
Source: Genomics Proteomics Bioinformatics. 2022 Nov 15;21(3):470–82. doi: 10.1016/j.gpb.2022.11.001 (PMC10787017; doi:10.1016/j.gpb.2022.11.001)
Supplement: Supplementary Table S19 — KEGG enrichment analysis of genes located in the inversion regions of Chr28 in sika deer [file mmc19.docx]

**Table S19** **KEGG enrichment analysis of genes located in the inversion regions of Chr28 in sika deer**

| **Pathway ID** | **Pathways** | **Gene number** | ***P* value** |
| --- | --- | --- | --- |
| ko00010 | Glycolysis / Gluconeogenesis | 3 | 0.01491436 |
| ko04115 | p53 signaling pathway | 3 | 0.0215871 |
| ko00640 | Propanoate metabolism | 2 | 0.02468348 |
| ko00620 | Pyruvate metabolism | 2 | 0.02901642 |
| ko00270 | Cysteine and methionine metabolism | 2 | 0.04232962 |
| ko04066 | HIF-1 signaling pathway | 3 | 0.04507269 |
