# Supplementary material for: Haplotype-resolved Genome of Sika Deer Reveals Allele-specific Gene Expression and Chromosome Evolution
Source: Genomics Proteomics Bioinformatics. 2022 Nov 15;21(3):470–82. doi: 10.1016/j.gpb.2022.11.001 (PMC10787017; doi:10.1016/j.gpb.2022.11.001)
Supplement: Supplementary Table S20 — Summary of the distribution of alleles on homologous chromosomes [file mmc20.docx]

**Table S20** **Summary of the distribution of alleles on homologous chromosomes**

| **Chromosome** | **Alleles number** | **The ratio of alleles in hap1** | **The ratio of alleles in hap2** |
| --- | --- | --- | --- |
| chr1.1 | 1177 | 60.08% | 68.51% |
| chr2.1 | 1077 | 64.15% | 73.32% |
| chr3.1 | 755 | 62.29% | 70.43% |
| chr4.1 | 465 | 59.54% | 71.76% |
| chr5.1 | 670 | 61.92% | 69.94% |
| chr6.1 | 544 | 66.83% | 69.30% |
| chr7.1 | 561 | 63.10% | 69.17% |
| chr8.1 | 443 | 66.42% | 73.10% |
| chr9.1 | 485 | 65.54% | 74.16% |
| chr10.1 | 272 | 58.87% | 65.70% |
| chr11.1 | 466 | 63.14% | 67.05% |
| chr12.1 | 285 | 59.25% | 70.37% |
| chr13.1 | 390 | 60.94% | 70.14% |
| chr14.1 | 234 | 61.90% | 68.82% |
| chr15.1 | 207 | 59.31% | 70.89% |
| chr16.1 | 363 | 62.69% | 67.85% |
| chr17.1 | 156 | 67.53% | 69.96% |
| chr18.1 | 223 | 58.68% | 62.64% |
| chr19.1 | 177 | 63.90% | 64.84% |
| chr20.1 | 400 | 62.21% | 68.73% |
| chr21.1 | 340 | 61.15% | 64.15% |
| chr22.1 | 206 | 57.38% | 62.42% |
| chr23.1 | 356 | 62.02% | 77.22% |
| chr24.1 | 299 | 64.16% | 75.89% |
| chr25.1 | 393 | 58.22% | 64.74% |
| chr26.1 | 113 | 59.16% | 64.57% |
| chr27.1 | 192 | 56.80% | 67.61% |
| chr28.1 | 344 | 62.43% | 68.94% |
| chr29.1 | 228 | 61.79% | 64.77% |
| chr30.1 | 141 | 54.86% | 60.78% |
| chr31.1 | 423 | 62.11% | 68.12% |
| chr32.1 | 149 | 51.38% | 58.43% |
| Total | 12,534 | 56.60% | 67.01% |
