# Supplementary material for: Haplotype-resolved Genome of Sika Deer Reveals Allele-specific Gene Expression and Chromosome Evolution
Source: Genomics Proteomics Bioinformatics. 2022 Nov 15;21(3):470–82. doi: 10.1016/j.gpb.2022.11.001 (PMC10787017; doi:10.1016/j.gpb.2022.11.001)
Supplement: Supplementary Table S21 — Summary of structural variation between two haplotypes [file mmc21.docx]

**Table S21** **Summary of structural variation between two haplotypes**

| **Chromosome** | **Deletion (bp)** | **Insertion (bp)** |
| --- | --- | --- |
| chr1.2 | 328,626 | 346,634 |
| chr2.2 | 398,150 | 376,123 |
| chr3.2 | 261,270 | 344,021 |
| chr4.2 | 346,986 | 504,552 |
| chr5.2 | 594,885 | 329,796 |
| chr6.2 | 351,027 | 253,937 |
| chr7.2 | 394,722 | 289,876 |
| chr8.2 | 430,847 | 399,687 |
| chr9.2 | 390,039 | 302,936 |
| chr10.2 | 432,347 | 281,222 |
| chr11.2 | 329,863 | 312,041 |
| chr12.2 | 311,695 | 293,950 |
| chr13.2 | 278,360 | 255,641 |
| chr14.2 | 360,012 | 197,931 |
| chr15.2 | 259,132 | 237,622 |
| chr16.2 | 210,905 | 258,110 |
| chr17.2 | 396,651 | 190,047 |
| chr18.2 | 333,998 | 281,309 |
| chr19.2 | 316,201 | 236,771 |
| chr20.2 | 185,293 | 166,645 |
| chr21.2 | 257,388 | 284,176 |
| chr22.2 | 181,738 | 216,284 |
| chr23.2 | 206,088 | 179,441 |
| chr24.2 | 257,077 | 186,579 |
| chr25.2 | 225,439 | 110,788 |
| chr26.2 | 199,779 | 267,930 |
| chr27.2 | 278,607 | 346,634 |
| chr28.2 | 257,983 | 376,123 |
| chr29.2 | 164,487 | 344,021 |
| chr30.2 | 178,177 | 504,552 |
| chr31.2 | 120,854 | 329,796 |
| chr32.2 | 161,195 | 253,937 |
